# Patient-reported outcome measures used in patients with primary sclerosing cholangitis: a systematic review

**DOI:** 10.1186/s12955-018-0951-6

**Published:** 2018-07-05

**Authors:** Fatima Isa, Grace M. Turner, Geetinder Kaur, Derek Kyte, Anita Slade, Tanya Pankhurst, Larissa Kerecuk, Thomas Keeley, James Ferguson, Melanie Calvert

**Affiliations:** 1 Public Health England, 5 St Philips Place, Birmingham, B3 2PW UK; 2 Centre for Patient Reported Outcomes Research (CPROR), Institute of Applied Health Research, University of Birmingham, Edgbaston, Birmingham, B15 2TT UK; 3 NIHR Birmingham Biomedical Research Centre, Birmingham, B15 2TT UK; 4 University Hospital Birmingham, Birmingham, B15 2TH UK; 5 Birmingham Children’s Hospital, Birmingham, B4 6NH UK; 6 PAREXEL International, Evergreen House North, 160 Euston Road, London, NW1 2DX UK

**Keywords:** Primary sclerosing cholangitis, Cholestasis, Patient reported outcome measures (PROMs), PROSPERO (Registration Number: CRD42016036544).

## Abstract

**Background:**

Primary Sclerosing Cholangitis (PSC) is a rare chronic, cholestatic liver condition in which patients can experience a range of debilitating symptoms. Patient reported outcome measures (PROMs) could provide a valuable insight into the impact of PSC on patient quality of life and symptoms. A previous review has been conducted on the quality of life instruments used in liver transplant recipients. However, there has been no comprehensive review evaluating PROM use or measurement properties in PSC patients’ to-date. The aim of the systematic review was to: (a) To identify and categorise which PROMs are currently being used in research involving the PSC population (b) To investigate the measurement properties of PROMs used in PSC.

**Methods:**

A systematic review of Medline, EMBASE and CINAHL, from inception to February 2018, was undertaken. The methodological quality of included studies was assessed using the Consensus-based Standards for selection of health Measurement Instruments (COSMIN) checklist.

**Results:**

Thirty-seven studies were identified, which included 36 different PROMs. Seven PROMs were generic, 10 disease-specific, 17 symptom-specific measures and 2 measures on dietary intake. The most common PROMs were the Short form-36 (SF-36) (*n* = 15) and Chronic liver disease questionnaire (CLDQ) (*n* = 6). Only three studies evaluated measurement properties, two studies evaluated the National Institute of Diabetes Digestive and Kidney Diseases Liver Transplant (NIDDK-QA) and one study evaluated the PSC PRO; however, according to the COSMIN guidelines, methodological quality was poor for the NIDDK-QA studies and fair for the PSC PRO study.

**Conclusion:**

A wide variety of PROMs have been used to assess health-related quality of life and symptom burden in patients with PSC; however only two measures (NIDDK-QA and PSC PRO) have been formally validated in this population. The newly developed PSC PRO requires further validation in PSC patients with diverse demographics, comorbidities and at different stages of disease; however this is a promising new measure with which to assess the impact of PSC on patient quality of life and symptoms.

**Electronic supplementary material:**

The online version of this article (10.1186/s12955-018-0951-6) contains supplementary material, which is available to authorized users.

## Background

Primary Sclerosing Cholangitis (PSC) is a chronic, cholestatic liver condition that results in inflammation and fibrosis that can involve the entire biliary tree [1]. PSC is a progressive disorder and can lead to cirrhosis, portal hypertension and liver failure [1].

Approximately 1 in 100,000 people in the general population is affected with PSC per year in Europe and the United States [2]. The disease occurs at any age, but is more prevalent in adults between the ages of 30–60 years and is more common in men than in women. Approximately 70–80% of patients with PSC have an associated inflammatory bowel disease (IBD) such as ulcerative colitis or Crohn’s disease [3]. Currently, there is no known licensed medication to prevent the progression of PSC, which if left untreated can result in increasing disability and even death [4]. In patients with end-stage PSC liver disease, the only therapeutic option currently available is a liver transplant [4].

Although overall disease progression can be slow, patients with PSC can experience a range of debilitating symptoms. In the early stage of the disease, symptoms include tiredness or fatigue. In more advanced cases, symptoms include pruritus, jaundice, abdominal pain, weight loss, fevers, hyperpigmentation, vitamin deficiencies and metabolic bone disease [5]; all of which can have a significant impact on health-related quality of life (HRQOL) [6, 7].

Increasingly in chronic diseases and terminal illness, it is recognised that maintaining HRQOL is an important consideration when the treatment is aimed at maintenance rather than a cure, or the treatment has a high level of toxicity [8]. Many of the current therapeutic interventions in PSC are aimed at managing symptoms. Measuring the impact of these interventions and preserving HRQOL is an important aspect of PSC care. This requires patient reported outcome measures (PROMs) that are sensitive enough to capture changes in HRQOL or symptoms over time.

Increasingly, PROMs use has demonstrated a positive contribution to clinical practice and research [9]. In clinical practice, aggregate level PROM data can help us to understand the burden of chronic medical conditions, identify health inequalities [10] and determine new areas for therapeutic interventions. They can also play a key role in benchmarking and audit. [11] At an individual patient level, PROMs can be used to monitor the response, adverse effects and benefits of treatments in routine practice, [12] facilitating communication between clinicians and patients regarding their HRQOL, symptom management and control [13–15].

A previous review investigating the quality of life (QOL) instruments used in liver transplant recipients has been conducted [16]. However, to date, no comprehensive review of PROMs used in PSC patients has been undertaken. There is a clear need to evaluate the measurement properties of the PROMs currently used in this population to determine the optimal measures for use in future research and routine care. Therefore the objectives of this systematic review were to: (a) identify and categorise PROMs currently used in research involving the PSC population; and (b) investigate their measurement properties, to help inform the selection of PROMs for use in future PSC research and routine practice.

## Methods

The following guidelines were used, where applicable, to inform the conduct and reporting of this study: (i) the Preferred Reporting Items for Systematic Reviews and Meta-Analyses (PRISMA) [17] guidance (see Additional file 1 for the PRISMA checklist), (ii) COnsensus based Standards for the selection of health Measurement INstruments (COSMIN) guidance [18] and (iii) the updated method guidelines for systematic reviews in Cochrane collaboration back review group [19]. The study was registered with PROSPERO (Registration Number: CRD42016036544).

### Search strategy

A systematic search was conducted on the following electronic databases: Medline, EMBASE and CINAHL from inception to 15 February 2018. The search terms “Primary sclerosing cholangitis” and “Patient reported outcome measures” were used, alongside synonyms and related terms (see Additional file 2 for the full search strategy). These terms were combined with the COSMIN search filters developed by VU University Medical Centre Amsterdam and University of Oxford (available on COSMIN website: http://www.cosmin.nl/). In addition, papers included in the full text review were subjected to a hand search of reference lists [20, 21].

### Inclusion criteria

Studies were eligible if:

- PROMs were included in the study meeting the FDA definition [22].
- Study participants were patients with PSC.

In addition:

c) Studies that evaluated at least one measurement property (i.e. reliability, validity, responsiveness, interpretability) were included in the COSMIN quality review.

No restriction was placed on age or gender of participants or language, publication date or country of origin of the study.

### Selection of studies

Two reviewers (FI/GT or GT/GK) independently screened studies according to their title and abstract to determine eligibility. Following this, the full text of potentially eligible studies was retrieved and screened independently by two independent reviewers (FI/GT or GT/GK). The protocol planned that discrepancies would be discussed with a third investigator (MG or DK or AS) to reach consensus; however, this was not required.

### Data extraction

The two independent reviewers (GT plus FI, GK or AS) independently extracted the data from each study using a predefined form (including study design and patient level characteristics). Information regarding each PROM was extracted, including: constructs, therapeutic area, domains, number of items, scoring method, recall period, administration, completion time, data collection, cost/permission and measurement properties (reliability, validity, responsiveness, interpretability).

### Content comparison of included PROMs

A summary of PROMs used in studies of PSC patients, including an overview of included domains and specific content was prepared. The PROMs were categorised according to their domains to facilitate comparison of the measures that have been used in PSC studies to-date.

### Quality assessment

The COSMIN checklist [23] was used to assess the methodological quality of studies that reported on the measurement properties of PROMs used in the study. Two reviewers (FI/GT or GT/AW) independently completed the COSMIN checklist. The protocol planned that discrepancies would be discussed with a third reviewer; however, this was not required. Each measurement property was scored according to the quality of reporting by the publication, using a four-point rating scale: ‘excellent’, ‘good’, ‘fair’ and ‘poor’. The methodological quality of each study was rated by taking the lowest score (worst score counts method) per domain. For example, if any of the items of the domain reliability was scored ‘poor’, the overall score for regarding the methodological quality of reliability was rated as ‘poor’.

### Evidence synthesis

Synthesis of measurement property evidence was performed using standardised criteria developed by Terwee 2011 [23]. The summary of the overall evidence of measurement properties of the PROMs was determined by the number of studies, the methodological quality of the studies, and consistency of the findings. Based on these factors the overall rating of a measurement property per PROM was ranked as “+” positive, “?” indeterminate or “-” negative and combined with an assessment of the overall level of supporting evidence (strong, moderate, limited, conflicting, unknown) as proposed by the Cochrane Back Review Group [24].

## Results

### Study selection

**Fig. 1:**
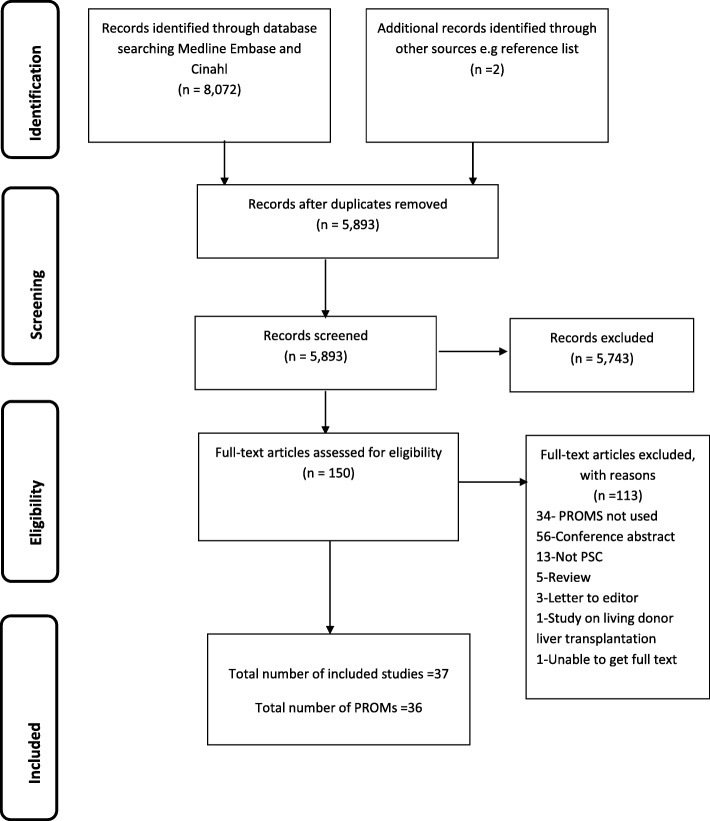
PRISMA flowchart describing the identification, selection and inclusion of studies on PROM assessment in Primary sclerosing Cholangitis

In total, 8074 studies were identified, 5893 remained after duplicate removal and 150 remained after reviewing titles and abstracts (Fig. 1). Following review of the 150 full texts, 37 studies, containing 36 different PROMs, were included.

Table 1 summarises the general characteristics of the included studies. The study designs included 17 cross-sectional studies, five randomised controlled trials (RCTs), four case-control studies, two validation study, two pilot study, two before and after study, one cost-effectiveness study, one case matched study, one longitudinal study, one cohort study and one retrospective case series study.

**Table 1:** Characteristics of included studies

Author (Year) *(Reference)*	Country	Study design	Sample size (PSC cases)	Mean age (SD) year	Gender (Male n %)	Disease stage	Mayo risk score / MELD Score	IBD (Yes/No (n (%))	LT (Yes/No (n (%))	PROM	Rationale for Assessment	PROM administration
Gavaler (1991) [66]	USA	Cross- sectional study	23 (23)	Quiescent group: 34.7 (6.2) Symptomatic group: 39.8 (1.6)	15 (65%)	Symptomatic UC: Mild: 7 (40%) Moderate: 8 (47%) Severe: 2 (13%)	NR	Yes (23 (100%))	Yes (23 (100%))	Study questionnaire: symptoms of UC	A	Postal & telephone
Gross (1999) [26]	USA	Before & after study	157 (92)	Total sample: 50 (10)	31 (34%)	NR	MRS: Mean 5.3	NR	Yes (157 (100%))	NIDDK-QA, pilot version NIDDKQA	A	Clinic
Kim (2000) [28]	USA	Validation study	96 (17)	45 (9.3)	7 (41%)	PSC undergoing LT: 17 (100%)	MRS: mean (SD) = −0.1(1.0)	NR	PSC patients undergoing LT: 17 (100%)	NIDDK-QA, SF- 36	D	Clinic
Bharucha (2000) [67]	USA	Pilot study	20 (20)	44 (11)	12 (60%)	Early stage (1–2): 10 (50%), Late stage (3–4): 10 (50%)	MRS: mean (SD) = 2.87 (0.95)	Yes (14 (70%))	No	Grading system fatigue & pruritus	B	Unclear
Younossi (2000) [38]	USA	Cross-sectional study	104 (29)	Total sample:: 55 (12)	Total sample 28 (97%)	NR	NR	NR	No	SF- 36, CLDQ	A	Unclear
Younossi (2001) [39]	USA	Cross-sectional study	353 (45)	Total sample: 54 (11)	Total sample 38 (30%)	Total sample: Child-pugh class: no cirrhosis: 47 (13%) class A: 43 (12%) class B-27 (8%) class C-4 (1%)	NR	NR	NR	SF-36, CLDQ	A	Clinic
Longworth (2003) [45]	England and Wales	Cost effectiveness study	347 (70)	NR	48 (69%)	NR	Of 41 patients MELD score median/IQR = 10/6–16	NR	Yes (45) 64%))	EuroQol EQ. 5D	C	Postal
Bjornsson (2004) [44]	England & Sweden	RCT	93 (20)	NR	13 (65%)	Cirrhosis: 5 (1%),Ludwig’s fibrosis score stage 1: 9 (44%), stage 2: 4(21%), stage 3:6(30%)	NR	Yes (16 (80%))	No	PGWB, FIS, BDI, GSRS, Rome ll modular QA	A	Postal
Ter Borg (2004) [36]	Netherlands	RCT	33 (11)	NR	10 (91%)	NR	NR	NR	No	VAS, FFSS, MFI	B	NR
Ter Borg (2005) [48]	Netherlands	Cross-sectional study	72 (27)	45 (NR)	19 (70%)	Cirrhosis: 15 (56%)	NR	Yes (2 (7%)	NR	VAS, FFSS, SF-36	A	NR
Olsson (2005) [33]	Sweden, Norway, Denmark	RCT	198 (198)	UDCA: 43.6(12.7) Placebo: 43.1 (11.2)	139 (70%)	NR	NR	Yes (168 (85%))	NR	SF- 36	B	Unclear
Gorgun (2005) [21]	USA	Case matched study	65 (65)	43.37 (11.2)	45 (69%)	NR	NR	Yes (65 (100%))	No	FPQ, CGQOL	A	
Mansour-Ghanaei (2006) [49]	Iran	RCT	34 (6)	Total sample: 53.97 (11.93)	NR	NR	NR	NR	NR	VAS	B	Unclear
Mayo (2007) [50]	USA	RCT	21 (4)	Total sample: 53.97 (11.93)	Total sample 5 (15%)	NR	^a^Total sample MELD mean (range): 11(6–24)	NR	NR	VAS, IDS-SR30	B	Unclear
Van os (2007) [52]	Netherlands	Cross-sectional study	92(37)	43.8(12.3)	24 (65%)	Cirrhosis: 5 (13.5%)	NR)	NR	NR	BDI, SADS	A	Postal
Tillman (2009) [37]	Germany	Cross-sectional study	511(13)	42 (NR)	NR	NR	NR	NR	NR	SF- 36, FIS, WHOQOL-BREF, HADS	A	In clinic
Ananthakrishnan (2010) [47]	USA	Case-control study	26 (26)	40.7 (14.8)	21 (80.8%)	NR	MELD score mean (range) 8 (6–20)	Yes (26(100%))	No	SIBDQ, HBI, UCAI	A	Outpatient clinic
Aberg (2012) [30]	Finland	Cross-sectional study	401 (56)	53 (9)	36 (64%)	NR	NR	NR	Yes (56 (100%))	15D, ad hoc questionnaire	A	Postal
Benito De Valle (2012) [29]	England & Sweden	Cross-sectional study	182 (182)	160 patients no LT: 50 (16)	112 (70%)	Small duct disease: 17 (11%), Liver cirrhosis: 12 (8%), Decompensated liver disease: 9 (6%)	MRS mean (SD): 0.34 (1.10)	Yes (126 (79%))	Yes (22 (12%))	SF-36, CLDQ, FIS, HADS	A	Postal
Hagstrom (2012) [68]	Sweden	Cross-sectional study	96 (96)	47 (13)	63 (66%)	Cases child pugh score of 10, significant fibrosis: 26 (27%), non-significant fibrosis: 70 (73%)	NR	Yes (73 (76%))	Yes (12 (12.5%))	LDH	A	Interview
Gulati (2013) [25]	USA	Cross-sectional study	40 (24)	Total sample: 11.6 (4.5)	17 (43%)	Total sample: Cirrhosis 22 (55%)	NR	Total sample: Yes (16 (65%))	No		A	Unclear
Block (2014) [69]	Norway & Sweden	Case-control study	48 (48)	NR	40 (83%)	NR	NR	48	Yes (IPAA: 11, IRA: 7)	OS	A	Scheduled follow up visit
Gotthardt (2014) [6]	Germany	Cross-sectional study	113 (113)	43.6 (14.2)	81 (71.7%)	NR	MRS n: low/intermediate/ high =48 (42%) / 25 (22%) / 5 (4%)	Yes (71 (63%))	NR	SF 36, PHQ-9	A	Postal
Hov (2014) [70]	Norway	Case-control study	240 (240)	NR	171 (71%)	NR	NR	Yes (183 (77%))	Yes (94 (39%))	Study questionnaire	A	Postal
Pavlides (2014) [34]	England	Retrospective case note review	40 (PSC-IPAA = 21 & PSC-UC = 19)	NR	31 (78%)	PSC-IPAA had dysplasia: 2 (5%)	NR	Yes (19 (47.5%))	No	OS, CGQOL, FSFI, IIEF	A	Postal
Raszeja-Wyszomirska (2014) [35]	Poland	Cross-sectional study	102 (102)	36 (12)	73 (72%)	Cirrhosis: 30 (29%)	NR	Yes (65 (64%))	NR	SF 36, PBC-40, PBC-27	A	Unclear
Cheung (2015) [32]	Canada	Cross-sectional study	162 (99)	46.1 (15.1)	50 (51%)	Cirrhosis: 47 (48%), Decompensated liver disease: 16 (16%)	NR	Yes (74)	No	SF-36, PBC-40, PHQ-9, LDQOL, SIBDQ, 10 peered-reviewed QA on emotional and psychosocial	A	Postal or clinic
Dyson (2015) [20]	USA	Cross-sectional study	40 (40)	51 (13)	31 (78%)	NR	NR	Yes (24 (60%))	NR	FIS, ESS, HADs, COMPASS	A	Postal
Eaton (2015) [71]	Canada & USA	Case-control study	1000 (1000)	NR	619 (72%)	NR	NR	Yes (741 ((74%))	Yes (450 ((45%))	HHQ	A	Postal or clinic
Haapamaki (2015) [31]	Finland	Cross-sectional study	341 (341)	43.3 (13.7)	183 (54%)	ERC-score mean (SD): 5.9 (3.4)	NR	Yes (237 (69.5%))	Yes (9 (2.6%))	15D, study questionnaire	A	ERC examination at the HUGH endoscopy unit
Kalaitzakis (2015) [27]	England and Sweden	Cross-sectional study	163 (163)	No LT: 50 (16)	No LT 122 (75%)	No LT Small-duct disease: 15 (10%), Diver cirrhosis: 11 (8%), Decompensated liver disease: 8 (6%)	No LT MRS: mean (SD) = 0.11(1.42)	No LT Yes (116 (71%))	Yes (19 (12%))	SF 36, SF-6D, CLDQ, study questionnaire	A, C	Unclear
Raszeja-Wyszomirska (2015) [41]	Poland	Cross-sectional study	33 (33)	35.3 (13.38)	11 (33%)	Cirrhosis: 6 (18%)	NR	Yes (22 (67%)	NR	SF 36, PBC-40, PBC-27	A	NR
Carbone (2017) [46]	Italy	Longitudinal study	227 (64)	50(11)	39 (66%)	NR	NR	NR	NR	EQ-5D	A	Clinic
Kempinska (2017) [40]	Poland	Cohort study	275 (275)	Median 55, range 28–90	182 (66%)	NR	NR	NR	NR	SF 36, PBC-40, PBC-27	A	NR
Kittanamongkolchai (2017) [51]	USA	Before and after study	13 (5)	46.4 (13.2)	1 (20%)	NR	NR	NR	NR	Pruritus numerical rating scale	B	Physician administered
Tabibian (2017) [42]	USA	Pilot study	16 (16)	40 (NR)	13 (81%)	All patients had stage 1–3 PSC	NR	13 (81%)	NR	FFSS, 5-D itch scale, CLDQ, SF-36	B	NR
Younossi (2017) [43]	USA	Validation study	102 (102)	44 (13)	33 (32%)	Cirrhosis: 37 (39%)	NR	67 (68%)	NR	PSC PRO, SF-36, CLDQ, PBC-40, 5-D Itch	D	ePRO website

*15D* 15-dimensional health-related quality of life measure, *5-D Itch* Five dimensions Itch, *BDI* Beck Depression Inventory, *CGQOL* Cleveland global quality of life questionnaire, *CLDQ* Chronic liver disease questionnaire, *COMPASS* Composite Autonomic Symptom Scale, *ESS* Epworth Sleepiness Scale, *EQ. 5D* EuroQol EQ. 5D, *FFSS* Fisk Fatigue Severity Scale, *FIS* Fatigue Impact Scale, *FSFI* Female Sexual Satisfaction Index, *GSRS* Gastrointestinal Symptom Rating Scale, *HADS* Hospital anxiety and depression scale, *HBI* Harvey-Bradshaw Index, *HHQ* Health Habits and History Questionnaires, *IBD* Inflammatory Bowel Disease, *IDS-SR30* 30-item Inventory of Depressive Symptomatology-self report, *IIEF* International index of erectile function, *LDH* Lifetime drinking history, *LDQOL* Liver Disease Quality of Life Questionnaire, *LT* Liver Transplant, *MELD* Model For End-Stage Liver Disease, *MFI* Multidimensional Fatigue Inventory, *MRS* Mayo Risk Score, *NIDDK-QA* National institute of diabetes and digestive and kidney disease liver transplant questionnaire, *NR* Not Reported, *OS* Oresland Scale, *PBC-40* Primary Biliary Cirrhosis, *PF* Pouch Function Questionnaire, *PGWB* Psychological general well-being index, *PHQ-9* Patient Health Questionnaire, *PSC PRO* Primary Sclerosing Cholangitis patient-reported outcome, *RCT* Randomised Controlled Trial, *SADS* Schedule for Affective Disorders and Schizophrenia, *SD* Standard Deviation, *SF-36* Short form 36, *SIBDQ* Short Inflammatory Bowel Disease Questionnaire, *UC* Ulcerative Colitis, *UCAI* UC Activity Index, *VAS* Visual Analogue Scale, *WHOQOL-BREF* World Health Organization Quality of Life assessment instrument

^a^Rationale for assessment: A; Burden (HRQOL /symptom) of disease, B: Effectiveness of treatment, C: Cost Effectiveness/Health Utilities, D:Validation of a Patient Reported Outcome Measure, (PROM)

Twenty seven of the 37 included studies used PROMs to examine the impact of PSC on patients and seven of these measured the effectiveness of treatments: one study evaluated the cost-effectiveness of liver transplantation, one study assessed health utilities and two were validation studies of the PROMs: the National Institute of Diabetes Digestive and Kidney Diseases Liver Transplant (NIDDK-QA) and the Primary Sclerosing Cholangitis Patient Reported Outcome (PSC PRO).

In total, 3742 patients with PSC were recruited to the included studies (sample size range *n* = 4–1000). All participants were adults, with the exception of one study [25] which included patients with the mean age of 11.6 years. Studies were heterogeneous in terms of population demographic characteristics. In the thirty-five studies that reported gender, the proportion of PSC patients who were males ranged from 15 to 97%. Five studies reported a relatively wide range of mean Mayo risk scores (− 0.1 to 2.87) for PSC patients, a score which estimates patient survival in PSC [6, 26–29]. Twenty-four studies described the proportion of IBD in PSC patients, ranging from 7 to 100%. In 12 studies, the percentage of PSC patients who had received a liver transplant ranged from 12 to 100%.

### Characteristics of PROMs

Characteristics of the 36 included PROMs are presented in Table 2. The most frequently used PROM was the Short Form 36 health survey (SF-36) (*n* = 15), followed by the Chronic Liver Disease Questionnaire (CLDQ) (*n* = 6) and the Primary Biliary Cirrhosis (PBC)-40 (*n* = 5). All other PROMs were used in ≤3 studies (Table 1).

**Table 2:** Characteristics of included PROMs

PROM	Construct	Therapeutic area	Domains	Total No. of items	Scoring method	Recall period	Administration	Completion time	Data collection^a^	Cost & permission^b^
15 D ©	HRQOL	Generic	Mobility,Vision,Hearing, Breathing, Sleeping, Eating, Speech, Elimination, Usual Activities, Mental function,Discomfort, symptoms, Depression, Distress, Vitality, Sexual Activity	15	1 to 5 levels	Present health status	Self-administered	5–10 min	PP	A, B
5-D Itch	Pruritus	Severity of symptoms	Duration, Degree, Direction, Disability, Distribution	5	0–5 (0 being least problematic and 5 most problematic)	Last 2 weeks	Self-administered	< 5 min	PP	Unknown
BDI	Psychological functioning (incl. coping)	Psychology/ Behaviour	Cognitive-affective, Somatic	21	Higher score = greater depression	Last 2 weeks including today	Self-administered/ Interviewer-administered	5–10 min	E, PP	B,D
CGQOL	HRQOL	Disease specific (IBD)	Unknown	3	0–1.0 (1 being the best)	Unknown	Unknown	Unknown	PP	Unknown
CLDQ	HRQOL	Digestive System Diseases	Abdominal symptoms, Fatigue, Systemic symptoms, Activity, Emotional function, Worry	29	Higher score = better QoL	Last two weeks	Self-administered	10 min	E, PP	B,D
COMPASS	Autonomic nervous system diseases	Signs and symptoms	Orthostatic intolerance, vasomotor, secretomotor, gastrointestinal, bladder and pupilometer	31	Higher score = higher autonomic symptom severity	In past year/ past 5 years	Self-administered	No information	PP	No information
EQ -5D	HRQOL	Generic	Mobility, Self-care, Usual activities, Pain/discomfort, Anxiety/depression	5 + VAS (20 cm)	Higher score = better QoL	Today	Interviewer-administeredProxy-ratedSelf-administered	A few minutes	E, PP, IVR, T	B,D
ESS	Sleep disorder	Signs and symptoms	Sleep	8	Higher score = higher sleepiness	Over recent times	Self-administered	2–3 min	E, PP	A,B
FFSS	HRQOL	Signs & symptoms	Fatigue	9	High score = higher fatigue	Past two weeks	Self-administered	< 5 min	E, PP	B,D
FIS	Symptoms of fatigue	Pathological Conditions, Signs and Symptoms	Cognitive functioning, Physical functioning, Psychosocial functioning	40	Lower score = less fatigue	Past four weeks	Self -administered	10 min	PP	A,B
FSFI	Signs and symptoms	Female Urogenital Diseases & Pregnancy	Desire, Arousal, Lubrication, Orgasm, Global satisfaction, Pain	19	Higher score = better functioning	During the past 4 weeks	Self-administered	Information not found	E, PP	C
Grading system for fatigue & pruritus	Fatigue and Pruritus	Severity of symptoms	Unknown	Unknown	Pruritus, grades 0 -no, 1-mild, 2- sleep interference,3- substantial sleep disturbance Fatigue, grade 0- no; 1- present, but no interference with activity; 2-extra rest required & activity limited 3- patient unable to work a full day.	Unknown	Unknown	Unknown	Unknown	Unknown
GSRS	Signs and symptoms	Signs & symptoms, Digestive system diseases	Abdominal pain syndrome, Reflux syndrome, Indigestion syndrome, Diarrhoea syndrome, Constipation syndrome	15	Lower score-better QoL	Last week	Self-administered	10 min	PP	B,D
HADS	Signs and symptoms	Nervous System Diseases Mental Disorders	Anxiety, Depression	14	Lower score = better QoL	In the past week	Self-administered	2–5 min	E, PP	C
HHHQ	Diet	Dietary habits	Patient demographics, Education, Medical surgical history and environmental exposure including dietary habits	370 questions	Unknown	Unknown	Self-report	Unknown	Unknown	Unknown
IDS-SRS 30	Signs and symptoms	Psychiatry/Psychology/Behaviour	Vegetative features, Cognitive changes, Mood disturbance, Endogenous symptoms, Anxiety symptoms	30(28 initial version)	Higher score = higher severity	Past 7 days	Clinical-rated, interviewer-administered, self-administered	10–15 min	E, IVR, PP	C
IIEF	HRQOL	Erectile Dysfunction	Erectile function, Orgasmic function, Sexual desire, Intercourse satisfaction, Overall satisfaction	15	Higher score = better QoL. Scores by dimension	Past 4 weeks	Self-administered	15 min	PP	B,D
LDH	Alcohol consumption patterns	Intake assessment	Consumption levels (quantity), frequency of use, variability in consumption, types of beverages, drinking pattern, solitary versus social drinking, time of the day alcohol consumption	Unclear	Scored by hand or calculator	Unknown	Unknown	20 min	Unknown	Cost nominal (copyright)
LDQOL 1.0	HRQOL	Digestive System Diseases	- Generic core SF-36v2 - Disease-targeted scales: Liver disease-related symptoms, Effects of liver disease, Concentration/Memory, Health distress, Sleep, Loneliness, Hopelessness, Stigma of liver disease, Sexual functioning/problems	72	Higher score = Better HRQOL.	The past 4 weeks; Presently (for few items)	Self-administered	18 (+/− 9) min	PP	D
MFI	Signs and symptoms	Pathological conditions, signs and symptoms	General fatigue, Physical fatigue, reduced activity, Reduced motivation, Mental fatigue	20	Lower score = better QoL	Lately	Self-administered	5 min	PP	B
NIDDK-QA	HRQOL	Patients undergoing Liver transplant	Liver disease symptoms, physical functioning, health satisfaction & overall well-being (OWB)	47	Higher scores indicate better QOL	Unknown	Unknown	Unknown	Unknown	Unknown
OS	Functional outcome	IPAA or IRA	Bowel movements, urgency, evacuation difficulties, soiling or seepage, perianal/stomal soreness, protective pad, dietary restrictions and social handicap	Unclear	best 0, worst 15	Unknown	Unknown	Unknown	Unknown	Unknown
PBC-27	HRQOL	Disease specific	Symptoms, Dryness,Itch, Fatigue, Cognitive, Emotional and Social	40	Higher scores = greater symptoms impact & poorerHRQOL.	Last four weeks	Self-completion	< 5 min	PP	Unknown
PBC-40	HRQOL	Disease specific	Other Symptoms domain, Itch, Fatigue, Cognitive, Social and Emotional	27	Higher scores = greater symptoms impact & poorerHRQOL.	Last four weeks	Self-completion	5 min	PP	Free access
PedsQL 4.0	HRQOL	Generic	Physical functioning, Emotional functioning, Social functioning,school functioning	21 to 23	Higher score = better QoL	Standard version: past one month. Acute version: past 7 days	Interviewer-administered Proxy-rated Self-administered	5 min	PP	A,B
PGWB	HRQOL	Generic	Anxiety, Depression mood, Positive well-being, Self-control, General health, Vitality	22	Higher score = better QoL	Standard version = past month/ acute version = last week/ last four weeks	Self-administered/Interviewer-administered	15 min	PP	
PHQ-9	Depression	Severity of depression	Nine questions on symptoms	10	Depression severity:1–4: None; 5–9: Mild; 10–14: Moderate, 15–19: Moderately severe, 20 to 27: Severe	over past 2 weeks	Self-completion	2 to 5 min	PP	Unknown
Pruritus numerical rating scale	Pruritus	Severity of symptoms	Unknown	Unknown	Numerical rating scale 0–10 (0 for having no symptoms and 10 for having the worst imaginable pruritus)	Unknown	Unknown	Unknown	Unknown	Unknown
PSC PRO	HRQOL	Disease specific	PSC symptoms, Physical function, Activities of Daily Living, Work Productivity, Role Function, Emotional Impact, Social/Leisure Impact, Q uality of Life, Total Impact of Symptoms	42	Module 1: 0–10 scale; Module 2 has 7 four item domains: 1–5 scale, summed within dmains and domain mean summed to give overall impact score	Module 1–24 h recall	Self-administered	7–15 min	E, PP	Unknown
Rome ll modular questionnaire	Symptoms	Functional bowel disorder	Esophageal symptoms, Gastroduodenal symptoms, Bowel symptoms, Abdominal pain symptoms, Biliary symptoms and Anorectal symptoms	Unknown	Unknown	Unknown	Unknown	Unknown	Unknown	Unknown
SADS	Signs and symptoms	Depression	Depressive mood and ideation, Endogenous (ie. Melancholic, vital or vegetative) features, Depressive syndrome, Suicidal ideation and behaviour	30	Unknown	Past week only	Unknown	Unknown	Unknown	Unknown
SF-36	HRQOL	Generic	Physical Functioning, Role-Physical, Bodily Pain, General Health,Vitality, Social Functioning, Role-Emotional,Mental Health	36	0 to 100, higher score = better health status	Standard version 4 weeks / Acute version 1 week	Self-administered/Interviewer-administered	5–10 min	E, C, IVR, T, PP	B
SF-6D	Utilities & Health states	Generic- preference based measure	Physical functioning, role limitation, social functioning, pain, mental health, vitality	Unknown	0.296-most severe problems 1.0-no problems	Unknown	Unknown	Unknown	Unknown	Unknown
SIBDQ	HRQOL	Digestive System Diseases	Bowel symptoms, systematic symptoms, Emotional function, Social function	10	1 to 7, higher score = better QOL	Last two weeks	Self-administered/Interviewer-administered	5 min	E, PP	D
VAS	Fatigue and Pruritus	Severity of symptoms	Fatigue, Energy, Pruritus	Pruritus: 10 cm line	Pruritus 0 -no pruritus / 10- worst pruritus imaginable	Right now	Self-administered	Vas: Fatigue < 2 min	PP	Free access
WHOQOL-BREF	HRQOL	Generic	Physical, Psychological, social relationship, Environment, + 2 overall QOL & general health status	26	Higher score = better QoL	Last 2 weeks	Interviewer-administered, self-administered	5 min self-administration, 15–20 min interviewer-administration	PP	D

*15 D* 15-dimensional health-related quality of life measure, *5-D Itch* Five dimensions Itch, BDI: Beck Depression Inventory, *CGQOL* Cleveland global quality of life questionnaire, *CLDQ* Chronic liver disease questionnaire, *COMPASS* Composite Autonomic Symptom Scale, *EQ. 5D* EuroQol EQ. 5D, *ESS* Epworth Sleepiness Scale, *FFSS* Fisk Fatigue Severity Scale, *FIS* Fatigue Impact Scale, *FSFI* Female Sexual Satisfaction Index, *GSRS* Gastrointestinal Symptom Rating Scale, *HADS* Hospital anxiety and depression scale, *HBI* Harvey-Bradshaw Index, *HHQ* Health Habits and History Questionnaires, *HRQOL* Health-related quality of life, *IBD* Irritable Bowel Syndrome, *IDS-SR30* 30-item Inventory of Depressive Symptomatology-self report, *IIEF* International index of erectile function, *LDH* Lifetime drinking history, *LDQOL* Liver Disease Quality of Life Questionnaire, *MFI* Multidimensional Fatigue Inventory, *NIDDK-QA* National institute of diabetes and digestive and kidney disease liver transplant questionnaire, *No*. Number, *OS* Oresland Scale, *PBC-40* Primary Biliary Cirrhosis, *PF* Pouch Function Questionnaire, *PGWB* Psychological general well-being index, *PHQ-9* Patient Health Questionnaire, *PSC PRO* Primary Sclerosing Cholangitis patient-reported outcome, *QoL* Quality of Life, *SADS* Schedule for Affective Disorders and Schizophrenia, *SF-36* Short form 36, *SIBDQ* Short Inflammatory Bowel Disease Questionnaire, *UCAI* UC Activity Index, *VAS* Visual Analogue Scale, *WHOQOL-BREF* World Health Organization Quality of Life assessment instrument

^a^PP: Paper & pen, E: E-version, IVR: Interactive Voice Response, T: Telephone, C: Computer

^b^A: Free access to academic/non-profitable research, B: Fees for commercial/pharmaceutical companies/academics, C: Free access to public domain, D: Contact author / licence / signature of a contract or agreement

There were seven generic measures including: the 15 Dimensional Health-Related Quality of Life Measure (15D ©) [30, 31]; SF-36® [6, 27–29, 32–43]; Short Form 6 health survey (SF-6D) [27]; Psychological General Well-being Index (PGWBI) [44]; Paediatric Quality of Life Inventory™ generic core scale (PedsQL™) [25]; EuroQOL (EQ. 5D) [37, 45, 46]; and the World Health Organization Quality of Life assessment instrument (WHOQOL-BREF) [37].

Ten disease-specific measures included: the Short form Liver Disease Quality of Life questionnaire (LDQOL 1.0) [32]; CLDQ [27, 29, 38, 39, 42, 43]; the NIDDK-QA [26, 28]; Rome II Modular Questionnaire; the Cleveland Global Quality of Life questionnaire (CGQOL) [34]; the Short Inflammatory Bowel Disease Questionnaire (SIBDQ) [32, 47]; Oresland scale; PSC PRO; [43] PBC-27 [35, 40, 41]; and PBC-40 [32, 35, 40, 41, 43].

The 17 symptom-specific PROMs included: the FIS [29, 37, 44]; Gastrointestinal Symptom Rating Scale (GSRS) [44]; Fisk Fatigue Severity Scale (FFSS) [36, 42, 48]; Multidimensional Fatigue Inventory (MFI) [48]; VAS [48–50]; the 5-Dimension Itch; [42, 43] the Pruritus numerical rating scale; [51] the Hospital Anxiety and Depression Scale (HADS) [29]; Beck Depression Inventory (BDI) [44, 52]; Inventory of Depressive Symptomatology (IDS) [50]; Patient Health Questionnaire (PHQ-9) [6, 32]; Schedule for Affective Disorders and Schizophrenia (SADS) [52]; the Female Sexual Functioning Index (FSFI) [34]; International Index of Erectile Function (IIEF) [34]; Epworth Sleepiness Scale (ESS); [21] and Composite Autonomic Symptom Scale 31 (COMPASS 31) [21].

Two other measures included: the Lifetime Drinking History (LDH) and Health Habits and History Questionnaires (HHHQ), which focused on alcohol consumption and dietary intake.

### Content comparison of included PROMs

The most frequent health domains (*n* = 6) included across the measures were: fatigue, pain, physical functioning, emotion, anxiety and general health.

Generic PROMs measured symptoms such as pain, physical functioning, emotion, mental health and depression. The disease- and symptom-specific PROMs targeted aspects surrounding gastro intestinal symptoms, such as abdominal pain, or gastroduodenal symptoms, sexual problems, somatic symptoms, depression, mood disturbance, and vegetative features (Additional file 3).

### Quality assessment

Only three studies investigated measurement properties for PROMs, two studies evaluated the NIDDK-QA [26, 28] and one study evaluated the PSC PRO [43].

For NIDDK-QA, one validation study [28] included 76 Primary Biliary Cirrhosis (PBC) and 17 PSC patients. A second study examined health status and QOL in patients with cholestatic disease before and after a liver transplant. In this study the NIDDK-QA questionnaire was administered to 65 Primary Biliary Cirrhosis and 92 PSC patients [26]. The PSC PRO validation study included 102 patients with PSC who completed the PSC PRO and four other questionnaires (SF-36, CLDQ, PBC-40 and 5-D Itch Scale) using an ePRO website [43]. The results of the validation studies are presented in Table 3 and summarised below.

**Table 3:** Results of measurement properties of NIDDK-QA

PROM (Author, Year)	Total sample size	PSC sample size	Domains	Test retest reliability (Pearson Correlation)	Internal consistency (Cronbach’s Alpha)
NIDDK-QA (Kim, 2000)	96	17	Liver symptoms men women	0.94	Men = 0.94, women =0.87
			Physical function	0.99	0.88
			Health satisfaction	0.82	NR
			Overall well being	0.83	0.91
				Time interval of 2 weeks	
NIDDK-QA (Gross, 1999)	157	92	Symptoms	NR	0.81 & 0.85
			Functioning	NR	0.82 & 0.88
			Index of General Affect (IGA)	NR	0.91 & 0.93
PSC PROM (Younossi, 2017)	102	Test retest *n* = 53 Internal consistency *n* = 155	PSC Symptoms	0.84	0.89
			Physical Function	0.83	0.91
			Activities of Daily Living	0.85	0.86
			Work Productivity	0.7	0.93
			Role Function	0.83	0.91
			Emotional Impact	0.82	0.91
			Social/Leisure Impact	0.8	0.93
			Quality of Life	0.79	0.94
			Total Impact of Symptoms	0.88	

*NIDDK-QA* National institute of diabetes and digestive and kidney disease liver transplant questionnaire, *PSC PRO* Primary Sclerosing Cholangitis Patient Reported Outcome

#### Internal consistency

All the validation studies, appropriately calculated Cronbach’s alpha to estimate reliability and internal consistency. Reported Cronbach’s Alpha ranged from 0.87 to 0.94 for the NIDDK-QA and 0.86 to 0.94 for the PSC PRO which suggests good internal consistency. Criteria defined by the COSMIN tool meant that for the NIDDK-QA the measurement properties were evaluated as ‘poor’ in methodological quality in both studies primarily because of small sample sizes and a lack of information regarding the proportion of missing items and how missing items were managed. The PSC PRO was rated as ‘fair’ due to the lack of explicit reporting of missing items and sample size for unidemensionality analysis.

#### Reliability

Kim et al. (2000) [28] assessed test-retest reliability of the NIDDK-QA by administering the measure on two separate occasions approximately 2 weeks apart in 19 patients. Although Pearson’s correlation was high at 0.80 (range 0.82 to 0.94), this measurement property was evaluated as ‘poor’ methodological quality due to the small sample size. For the PSC PRO, 53 patients completed the PSC PRO a second time within 3 months and correlations between administrations was high (range 0.70–0.88). The reliability of the PSC PRO was rated as ‘fair’ due to this length of time between administrations.

#### Validity

Kim et al. (2000) [28] assessed concurrent validity, by investigating the correlation between the NIDDK-QA and SF-36. The authors postulated that observed correlations between theoretically related domains such as physical function and health satisfaction (*r* = 0.86 and 0.72 respectively) demonstrated concurrent validity of the tool. However, this measurement property was also evaluated with ‘poor’ methodological quality owing to the absence of details regarding the measurement properties of the comparator scale (SF-36) in this population, and issues with sample size and missing data.

Kim et al. (2000) [28] also measured discriminant validity and information on the significant differences in the item and domain level scores of NIDDK-QA reported. Again, this property was evaluated with ‘poor’ methodological quality, secondary to issues regarding sample size, proportion and handling of missing data.

For the PSC PRO, 26 PSC patients enrolled in cognitive interviews for assessment of content validity, which was rated as ‘excellent’ according to the COSMIN checklist. An external validation cohort of 102 patients completed the PSC PRO along with SF-36, CLDQ, PBC-40 and 5-D Itch Scale; all correlations were statistically significant. The structural validity measurement property was rated as ‘fair’ due to the sample size in relation to the number of items.

### Evidence synthesis

Both NIDDK-QA studies reported limited information regarding internal consistency, reliability and validity (concurrent and discriminant). Using the COSMIN guidance these properties were rated as indeterminate due to the poor methodological ratings of both studies (Tables 4 and 5) (Additional file 4) [23]. The PSC PRO study [43] had higher methodological quality compared to the NIDDK-QA studies; however, as there was only one study the level of evidence is limited.

**Table 4:** Methodological quality of each study per measurement property and PROM

Author (Year)	PROM	Internal consistency	Test-retest reliability	Measurement error	Content validity	Structural validity	Hypothesis testing	Criterion validity	Cross structural validity
							Discriminant validity	Concurrent validity	
Kim (2000)	NIDDK-QA	Poor	Poor	NR	NR	NR	Poor	Poor	NR
Gross (1999)	NIDDK-QA	Poor	NR	NR	NR	NR	NR	NR	NR
Younossi, (2017)	PSC PROM	Fair	Fair	NR	Excellent	Fair	NR	NR	NR

*NIDDK-QA* National institute of diabetes and digestive and kidney disease liver transplant questionnaire; PSC PRO: Primary Sclerosing Cholangitis Patient Reported Outcome

**Table 5:** Quality of measurement properties

PROM	Internal consistency	Test-retest reliability	Measurement error	Content validity	Structural validity	Hypothesis testing	Criterion validity	Responsiveness
						Discriminant validity	Concurrent validity	
NIDDK-QA	?	?	NR	NR	NR	?	?	NR
PSC PROM	+	+	NR	+	+	NR	NR	NR

Level of evidence (COSMIN): +++ or --- ‘Strong’ Consistent findings in multiple studies of good methodological quality, ++ or – ‘Moderate’ Consistent findings in multiple studies is fair, + or – ‘Limited’ One study of fair methodological quality, +/− ‘Conflicting’ Findings are conflicting,? ‘Unknown’ Studies of poor methodological quality. *NIDDK-QA* National institute of diabetes and digestive and kidney disease liver transplant questionnaire, *PSC PRO* Primary Sclerosing Cholangitis Patient Reported Outcome

## Discussion

This review identified a total of 37 studies assessing 36 different PROMs used in patients with PSC; however, only one of these tools was specifically developed for the PSC population in accordance with FDA guidelines. The rationale for PROM utilization in the included studies varied. Most studies sought to measure the burden of the disease using constructs such as HRQOL and symptom severity; however, some studies examined the effectiveness of treatment, cost effectiveness and health utility. No studies researched the use of real-time monitoring of PROMs to directly inform PSC patient care in a routine clinical setting. Only three studies evaluated the measurement properties of PROMs in PSC patients: two studies evaluated the NIDDK-QA [26, 28] and one study evaluated the PSC PRO [43]. Currently, due to weakness in the methodological quality, there is limited evidence to support the use of these PROMs in the PSC population; however the PSC PRO is a promising new measure designed with patient input which requires further validation.

Clinicians or researchers wishing to use PROMs in PSC patients may consider use of both generic and disease specific measures. Choice of measurement selection should be informed through consideration on psychometric properties and patient input [53]. Generic measures such as the SF-36, although not formally validated in PSC patients, are widely used and allow comparison of the burden of PSC with other chronic disease, whilst the EQ-5D and SF-6D may be used to provide estimates of health utility to inform cost-effectiveness analysis [54]. Use of the PSC PRO will provide a more detailed assessment of symptoms and impact of symptoms relevant to PSC patients and help identify patients with varying disease severity [43, 55].

Although the PSC PRO has been developed with input from patients with and without IBD, questions focused on IBD symptoms appear fairly limited. This is important to note since 70–80% of PSC patients have co-existent IBD, most frequently ulcerative colitis [3]. This is a long term comorbidity and can occur even after a liver transplant [56]. The clinical course for patients with PSC and concomitant IBD can be different when compared to IBD or PSC alone [57]. PSC-IBD patients have higher incidence of rectal sparing, colorectal neoplasia, pouchitis following ileal pouch anal anastomosis (IPAA), pancolitis, and an overall poorer prognosis when compared to patients with IBD alone [57, 58]. Thus, PSC-IBD patients have additional symptoms and burdens that impact on activities of daily living with the consequential impact on HRQOL [59]. Additional use of an IBD measure such as the IBS-QOL may therefore be warranted [60].

Following further validation, the PSC PRO has potential for use in a number of ways to inform PSC patient care. The PRO may be used in clinical trials to assess the impact of new treatments or be used at the individual patient level in routine clinical practice to facilitate shared decision making and tailor care to individual patient needs. This approach has been highly successful in other settings such as cancer where routine monitoring using ePROs reduced emergency room admissions by 7%, hospital admissions by 4%, helped patients stay on treatment longer, improved patient quality of life by 31% and increased survival on average by 5 months at low cost [61, 62].

### Strengths and limitations

This study is the first to undertake a systematic review of PROMs used in PSC, in accordance with the PRISMA [63] and COSMIN guidelines [64]. The use of COSMIN criteria has permitted a structured and comprehensive evaluation of the identified measures. However, the NIDDK QA studies evaluated in this review were carried out before the COSMIN guidance was available and at the time of publication the level and detail of reporting may have been deemed acceptable at that time. Another important consideration for research studies or clinical trials in rare diseases such as PSC are the small study populations. When guidelines such as COSMIN judge the quality of the methodology on sample sizes, it can make it more difficult to demonstrate sound methodological quality when there are only small numbers of patients available for recruitment and validation of PROs [65]. The use of international multi-centred studies may be one approach to overcome the small numbers available in studies that aim to evaluate and develop PROs for use in PSC in future studies.

## Conclusion

In conclusion, a wide variety of PROMs are used to assess HRQOL and symptom burden in patients with PSC, but none have undergone comprehensive and extensive validation in this patient group. The PSC PRO is a promising new measure to assess symptoms and symptom impact in PSC patients; however further validation work is required. Collection of PROs in PSC patients can provide valuable information in a research setting and routine clinical practice to improve PSC patient care.

## Additional files

Additional file 1: PRISMA checklist. (DOCX 62 kb)

Additional file 2: Medline search strategy, (DOCX 42 kb)

Additional file 3: Content comparison. (DOCX 52 kb)

Additional file 4: Cosmin checklist. (DOCX 22 kb)

## Abbreviations

15D: 15 Dimensional health-related quality of life measure
5-D Itch: Five dimensional itch
BDI: Beck depression inventory
CGQOL: Cleveland global quality of life questionnaire
CLDQ: Chronic liver disease questionnaire
COMPASS 31: Composite autonomic symptom scale 31
COSMIN: Consensus-based standards for selection of health measurement instruments
EQ 5D: EuroQOL
ESS: Epworth sleepiness scale
FDA: Food and Drug Administration
FFSS: Fisk fatigue severity scale
FIS: Fatigue impact scale
FSFI: Female sexual functioning index
GSRS: Gastrointestinal symptom rating scale
HADS: Hospital anxiety and depression scale
HHHQ: Health habits and history questionnaires
HRQOL: Health-related quality of life
IBD: Inflammatory bowel disease
IDS: Inventory of depressive symptomatology
IIEF: International index of erectile function
LDH: Lifetime drinking history
LDQOL 1.0: Short form liver disease quality of life questionnaire
MFI: Multidimensional fatigue inventory
NIDDK-QA: National Institute of Diabetes Digestive and Kidney Diseases Liver Transplant
PBC-27: Primary biliary cirrhosis
PBC-40: Primary biliary cirrhosis
PedsQL: Paediatric Quality of Life Inventory generic core scale
PGWBI: Psychological General Well-being Index
PHQ-9: Patient Health Questionnaire
PROMs: Patient-reported outcome measures
PSC PRO: Primary sclerosing cholangitis patient reported outcome
PSC: Primary sclerosing cholangitis
SADS: Schedule for affective disorders and schizophrenia
SF-36: Short Form 36 health survey
SF-6D: Short Form 6 health survey
SIBDQ: Short inflammatory bowel disease questionnaire
VAS: Visual analogue scale
WHOQOL-BREF: World Health Organization Quality of Life assessment instrument

## References

[1] Williamson KD, Chapman RW (2015). Primary sclerosing cholangitis: a clinical update. Br Med Bull.

[2] Primary Sclerosing Cholangitis [http://rarediseases.org/rare-diseases/primary-sclerosing-cholangitis/].

[3] Ponsioen C (2013). Diagnosis, prognosis, and Management of Primary Sclerosing Cholangitis. Gastroenterol Hepatol.

[4] Singh S, Talwalkar JA (2013). Primary Sclerosing cholangitis: diagnosis, prognosis, and management. Clin Gastroenterol Hepatol.

[5] Eaton JE, Talwalkar JA, Lazaridis KN, Gores GJ, Lindor KD: Pathogenesis of primary Sclerosing cholangitis and advances in diagnosis and management. Gastroenterology 2013, 145(3):10.1053/j.gastro.2013.1006.1052. 10.1053/j.gastro.2013.06.052 PMC3815445 23827861

[6] Gotthardt DN, Rupp C, Bruhin M, Schellberg D, Weiss KH, Stefan R, Donnerstag N, Stremmel W, Lowe B, Juenger J (2014). Pruritus is associated with severely impaired quality of life in patients with primary sclerosing cholangitis. Eur J Gastroenterol Hepatol.

[7] De Valle MB, Rahman M, Lindkvist B, Bjornsson E, Chapman RW, Kalaitzakis E (2010). Fatigue in patients with primary sclerosing cholangitis: an international survey study in two population-based patient cohorts. Gastroenterology.

[8] Phillips R, Gandhi M, Cheung YB, Findlay MP, Win KM, Hai HH, Yang JM, Lobo RR, Soo KC, Chow PKH (2015). Summary scores captured changes in subjects' QoL as measured by the multiple scales of the EORTC QLQ-C30. J Clin Epidemiol.

[9] Deshpande PR, Rajan S, Sudeepthi BL, Abdul Nazir CP (2011). Patient-reported outcomes: a new era in clinical research. Perspect Clin Res.

[10] Spiegel BMR (2013). Patient-reported outcomes in gastroenterology: clinical and research applications. J Neurogastroenterol Motility.

[11] Calvert M, Thwaites R, Kyte D, Devlin N (2015). Putting patient-reported outcomes on the 'Big data road Map'. J R Soc Med.

[12] Black N (2013). Patient reported outcome measures could help transform healthcare. BMJ.

[13] Velikova G, Booth L, Smith AB, Brown PM, Lynch P, Brown JM, Selby PJ (2004). Measuring quality of life in routine oncology practice improves communication and patient well-being: a randomized controlled trial. J Clin Oncol.

[14] Detmar SB, Muller MJ, Schornagel JH, Wever LD, Aaronson NK (2002). Health-related quality-of-life assessments and patient-physician communication: a randomized controlled trial. JAMA.

[15] Hilarius DL, Kloeg PH, Gundy CM, Aaronson NK (2008). Use of health-related quality-of-life assessments in daily clinical oncology nursing practice: a community hospital-based intervention study. Cancer.

[16] Jay CL, Butt Z, Ladner DP, Skaro AI, Abecassis MM (2009). A review of quality of life instruments used in liver transplantation. J Hepatol.

[17] Moher D, Liberati A, Tetzlaff J, Altman DG (2009). Preferred reporting items for systematic reviews and meta-analyses: the PRISMA statement. The BMJ.

[18] Mokkink LB, Terwee CB, Patrick DL, Alonso J, Stratford PW, Knol DL, Bouter LM, de Vet HCW (2010). The COSMIN checklist for assessing the methodological quality of studies on measurement properties of health status measurement instruments: an international Delphi study. Qual Life Res.

[19] Furlan AD, Malmivaara A, Chou R, Maher CG, Deyo RA, Schoene M, Bronfort G, van Tulder MW: 2015 updated method guideline for systematic reviews in the Cochrane back and neck group. Spine (Phila Pa 1976) 2015, 40(21):1660–1673. 10.1097/BRS.0000000000001061 26208232

[20] Dyson JK, Elsharkawy AM, Lamb CA, Al-Rifai A, Newton JL, Jones DE, Hudson M (2015). Fatigue in primary sclerosing cholangitis is associated with sympathetic over-activity and increased cardiac output. Liver Int.

[21] Gorgun E, Remzi FH, Manilich E, Preen M, Shen B, Fazio VW (2005). Surgical outcome in patients with primary sclerosing cholangitis undergoing ileal pouch-anal anastomosis: a case-control study. Surgery.

[22] U. S. Department of Health, human services F. D. A. Center for Drug Evaluation Research, U. S. Department of Health, human services F. D. A (2009). Center for Biologics Evaluation Research, U. S. Department of Health, human services F. D. A. Center for Devices Radiological Health: guidance for industry: patient-reported outcome measures: use in medical product development to support labeling claims.

[23] Terwee CB (2010). Consensus-based standards for the selection of health measurement instruments checklist.

[24] Furlan AD, Pennick V, Bombardier C, van Tulder M: 2009 updated method guidelines for systematic reviews in the Cochrane back review group. Spine (Phila Pa 1976) 2009, 34(18):1929–1941. 10.1097/BRS.0b013e3181b1c99f 19680101

[25] Gulati R, Radhakrishnan KR, Hupertz V, Wyllie R, Alkhouri N, Worley S, Feldstein AE (2013). Health-related quality of life in children with autoimmune liver disease. J Pediatric Gastroenterol Nutri.

[26] Gross CR, Malinchoc M, Ray Kim W, Evans RW, Wiesner RH, Petz JL, Crippin JS, Klintmalm GB, Levy MF, Ricci P (1999). Quality of life before and after liver transplantation for cholestatic liver disease. Hepatology.

[27] Kalaitzakis E, De Valle MB, Rahman M, Lindkvist B, Bjornsson ES, Chapman RW, Kontodimopoulos N. Mapping chronic liver disease questionnaire (CLDQ) scores onto SF-6D utility values in patients with primary sclerosing cholangitis: Results from a population-based cohort study. Gastroenterology. 2014;1:S–738.

[28] Kim WR, Lindor KD, Malinchoc M, Petz JL, Jorgensen R, Dickson ER (2000). Reliability and validity of the NIDDK-QA instrument in the assessment of quality of life in ambulatory patients with cholestatic liver disease. Hepatology.

[29] Benito de Valle M, Rahman M, Lindkvist B, Bjornsson E, Chapman R, Kalaitzakis E: Factors that reduce health-related quality of life in patients with primary sclerosing cholangitis. Clin Gastroenterol Hepatol 2012, 10(7):769–775.e762. 10.1016/j.cgh.2012.01.025 22343690

[30] Aberg F, Hockerstedt K, Roine RP, Sintonen H, Isoniemi H (2012). Influence of liver-disease etiology on long-term quality of life and employment after liver transplantation. Clin Transpl.

[31] Haapamaki J, Sintonen H, Barner-Rasmussen N, Farkkila M (2014). Health-related quality of life among patients with primary sclerosing cholangitis. J Crohn's Colitis.

[32] Cheung AC, Patel H, Meza-Cardona J, Cino M, Sockalingam S, Hirschfield GM: Factors that influence health-related quality of life in patients with primary Sclerosing cholangitis. Dig Dis Sci 2016;61(6):1692–9. 10.1007/s10620-015-4013-1 26743764

[33] Olsson R, Boberg KM, de Muckadell OS, Lindgren S, Hultcrantz R, Folvik G, Bell H, Gangsoy-Kristiansen M, Matre J, Rydning A (2005). High-dose ursodeoxycholic acid in primary sclerosing cholangitis: a 5-year multicenter, randomized, controlled study. Gastroenterology.

[34] Pavlides M, Cleland J, Rahman M, Christian A, Doyle J, Gaunt R, Travis S, Mortensen N, Chapman R (2014). Outcomes after ileal pouch anal anastomosis in patients with primary sclerosing cholangitis. J Crohn's Colitis.

[35] Raszeja-Wyszomirska J, Wunsch E, Krawczyk M, Rigopoulou EI, Bogdanos D and Milkiewicz P. Prospective evaluation of PBC-specific health-related quality of life questionnaires in patients with primary sclerosing cholangitis. Liver Int. 2015;35(6):1764–71. 10.1111/liv.12730 25388280

[36] ter Borg PC, van Os E, van den Broek WW, Hansen BE, van Buuren HR (2004). Fluvoxamine for fatigue in primary biliary cirrhosis and primary sclerosing cholangitis: a randomised controlled trial [ISRCTN88246634]. BMC Gastroenterol.

[37] Tillmann HL, Wiese M, Braun Y, Wiegand J, Tenckhoff S, Mossner J, Manns MP, Weissenborn K (2011). Quality of life in patients with various liver diseases: patients with HCV show greater mental impairment, while patients with PBC have greater physical impairment. J Viral Hepat.

[38] Younossi ZM, Kiwi ML, Boparai N, Price LL, Guyatt G (2000). Cholestatic liver diseases and health-related quality of life. Am J Gastroenterol.

[39] Younossi ZM, Boparai N, Price LL, Kiwi ML, McCormick M, Guyatt G (2001). Health-related quality of life in chronic liver disease: the impact of type and severity of disease. Am J Gastroenterol.

[40] Kempinska-Podhorodecka A, Milkiewicz M, Jabłonski D, Milkiewicz P, Wunsch E (2017). ApaI polymorphism of vitamin D receptor affects health-related quality of life in patients with primary sclerosing cholangitis. PLoS One.

[41] Raszeja-Wyszomirska J, Kucharski R, Zygmunt M, Safranow K, Miazgowski T (2015). The impact of fragility fractures on health-related quality of life in patients with primary sclerosing cholangitis. Hepat Mon.

[42] Tabibian JH, Gossard A, El-Youssef M, Eaton JE, Petz J, Jorgensen R, Enders FB, Tabibian A, Lindor KD (2017). Prospective clinical trial of rifaximin therapy for patients with primary sclerosing cholangitis. Am J Ther.

[43] Younossi ZM, Afendy A, Stepanova M, Racila A, Nader F, Gomel R, Safer R, Lenderking WR, Skalicky A, Kleinman L et al: Development and validation of a primary sclerosing cholangitis-specific patient-reported outcomes instrument: the PSC PRO. Hepatology (Baltimore*,* Md) 2017. 10.1002/hep.29664. 10.1002/hep.29664 29152767

[44] Bjornsson E, Simren M, Olsson R, Chapman RW (2004). Fatigue in patients with primary sclerosing cholangitis. Scand J Gastroenterol.

[45] Longworth L, Young T, Buxton MJ, Ratcliffe J, Neuberger J, Burroughs A, Bryan S, Team CP (2003). Midterm cost-effectiveness of the liver transplantation program of England and Wales for three disease groups. Liver Transpl.

[46] Carbone M, Cristoferi L, Cortesi PA, Rota M, Ciaccio A, Okolicsanyi S, Gemma M, Scalone L, Cesana G, Fabris L (2018). Optimising the clinical strategy for autoimmune liver diseases: principles of value-based medicine. Biochim Biophys Acta.

[47] Ananthakrishnan AN, Beaulieu DB, Naik AS, Zadvornova Y, Skaros S, Johnson K, Perera LP, Issa M, Binion DG, Saeian K (2009). Does primary sclerosing cholangitis impact quality of life in patients with inflammatory bowel disease?. Gastroenterology.

[48] ter Borg PC, Fekkes D, Vrolijk JM, van Buuren HR (2005). The relation between plasma tyrosine concentration and fatigue in primary biliary cirrhosis and primary sclerosing cholangitis. BMC Gastroenterol.

[49] Mansour-Ghanaei F, Taheri A, Froutan H, Ghofrani H, Nasiri-Toosi M, Bagherzadeh AH, Farahvash MJ, Mirmomen S, Ebrahimi-Dariani N, Farhangi E (2006). Effect of oral naltrexone on pruritus in cholestatic patients. World J Gastroenterol.

[50] Mayo MJ, Handem I, Saldana S, Jacobe H, Getachew Y, Rush AJ (2007). Sertraline as a first-line treatment for cholestatic pruritus. Hepatology.

[51] Kittanamongkolchai W, El-Zoghby ZM, Eileen Hay J, Wiesner RH, Kamath PS, LaRusso NF, Watt KD, Cramer CH, Leung N (2017). Charcoal hemoperfusion in the treatment of medically refractory pruritus in cholestatic liver disease. Hepatol Int.

[52] van Os E, van den Broek WW, Mulder PGH, ter Borg PCJ, Bruijn JA, van Buuren HR (2007). Depression in patients with primary biliary cirrhosis and primary sclerosing cholangitis. J Hepatol.

[53] Haywood KL, Wilson R, Staniszewska S, Salek S (2016). Using PROMs in healthcare: who should be in the driving seat-policy makers, health professionals, methodologists or patients?. Patient.

[54] Whitehead SJ, Ali S (2010). Health outcomes in economic evaluation: the QALY and utilities. Br Med Bull.

[55] Martin LM, Sheridan MJ, Younossi ZM (2002). The impact of liver disease on health-related quality of life: a review of the literature. Current Gastroenterol Rep.

[56] Joo M, Abreu-e-Lima P, Farraye F, Smith T, Swaroop P, Gardner L, Lauwers GY, Odze RD (2009). Pathologic features of ulcerative colitis in patients with primary sclerosing cholangitis: a case-control study. Am J Surg Pathol.

[57] Loftus EV, Harewood GC, Loftus CG, Tremaine WJ, Harmsen WS, Zinsmeister AR, Jewell DA, Sandborn WJ (2005). PSC-IBD: a unique form of inflammatory bowel disease associated with primary sclerosing cholangitis. Gut.

[58] Penna C, Dozois R, Tremaine W, Sandborn W, LaRusso N, Schleck C, Ilstrup D (1996). Pouchitis after ileal pouch-anal anastomosis for ulcerative colitis occurs with increased frequency in patients with associated primary sclerosing cholangitis. Gut.

[59] Achleitner U, Coenen M, Colombel J-F, Peyrin-Biroulet L, Sahakyan N, Cieza A (2012). Identification of areas of functioning and disability addressed in inflammatory bowel disease-specific patient reported outcome measures. J Crohn's Colitis.

[60] Lee J, Lee EH, Moon SH (2016). A systematic review of measurement properties of the instruments measuring health-related quality of life in patients with irritable bowel syndrome. Qual Life Res.

[61] Basch E, Deal AM, Dueck AC, Scher HI, Kris MG, Hudis C, Schrag D (2017). Overall survival results of a trial assessing patient-reported outcomes for symptom monitoring during routine cancer treatment. JAMA.

[62] Basch E, Deal AM, Kris MG, Scher HI, Hudis CA, Sabbatini P, Rogak L, Bennett AV, Dueck AC, Atkinson TM (2016). Symptom monitoring with patient-reported outcomes during routine cancer treatment: a randomized controlled trial. J Clin Oncol.

[63] Moher D, Shamseer L, Clarke M, Ghersi D, Liberati A, Petticrew M, Shekelle P, Stewart LA (2015). Preferred reporting items for systematic review and meta-analysis protocols (PRISMA-P) 2015 statement. Syst Rev.

[64] Terwee CB (2011). Protocol for systematic reviews of measurement properties.

[65] A decade of innovation in rare diseases [http://www.phrma.org/sites/default/files/pdf/PhRMA-Decade-of-Innovation-Rare-Diseases.pdf].

[66] Gavaler J, Delemos B, Belle SH, Heyl AE, Tarter RE, Starzl TE, Gavaler C, Van Thiel DH (1991). Ulcerative colitis disease activity as subjectively assessed by patient-completed questionnaires following orthotopic liver transplantation for sclerosing cholangitis. Dig Dis Sci.

[67] Bharucha AE, Jorgensen R, Lichtman SN, LaRusso NF, Lindor KD (2000). A pilot study of pentoxifylline for the treatment of primary sclerosing cholangitis. Am J Gastroenterol.

[68] Hagstrom H, Stal P, Stokkeland K, Bergquist A (2012). Alcohol consumption in patients with primary sclerosing cholangitis. World J Gastroenterol.

[69] Block M, Jorgensen KK, Oresland T, Lindholm E, Grzyb K, Cvancarova M, Vatn MH, Boberg KM, Borjesson L (2014). Colectomy for patients with ulcerative colitis and primary sclerosing cholangitis - what next?. J Crohn's Colitis.

[70] Hov JR (2014). Effects of coffee consumption, smoking, and hormones on risk for primary sclerosing cholangitis. Clin Gastroenterol Hepatol.

[71] Eaton JE, Juran BD, Atkinson EJ, Schlicht EM, Xie X, de Andrade M, Lammert CS, Luketic VA, Odin JA, Koteish AA (2015). A comprehensive assessment of environmental exposures among 1000 north American patients with primary sclerosing cholangitis, with and without inflammatory bowel disease. Aliment Pharmacol Ther.

