# Supplementary material for: Patient-reported outcome measures used in patients with primary sclerosing cholangitis: a systematic review
Source: Health Qual Life Outcomes. 2018 Jul 5;16:133. doi: 10.1186/s12955-018-0951-6 (PMC6034220; doi:10.1186/s12955-018-0951-6)
Supplement: Supplementary file 2 — Medline search strategy, (DOCX 42 kb) [file 12955_2018_951_MOESM2_ESM.docx]

**Additional file 2 Medline Search strategy**

| **#** | **Searches** |
| --- | --- |
| 1 | (instrumentation or methods).sh. |
| 2 | exp Psychometrics/ |
| 3 | psychometr*.ti,ab. |
| 4 | (clinimetr* or clinometr*).tw. |
| 5 | exp "Outcome Assessment (Health Care)"/ |
| 6 | outcome assessment.ti,ab. |
| 7 | outcome measure*.tw. |
| 8 | exp Observer Variation/ |
| 9 | observer variation.ti,ab. |
| 10 | exp Health Status Indicators/ |
| 11 | exp "Reproducibility of Results"/ |
| 12 | reproducib*.ti,ab. |
| 13 | exp Discriminant Analysis/ |
| 14 | (reliab* or unreliab* or valid* or coefficient or homogeneity or homogeneous or "internal consistency").ti,ab. |
| 15 | (cronbach* and (alpha or alphas)).ti,ab. |
| 16 | (item and (correlation* or selection* or reduction*)).ti,ab. |
| 17 | (agreement or precision or imprecision or "precise values" or test-retest).ti,ab. |
| 18 | (test and retest).ti,ab. |
| 19 | (reliab* and (test or retest)).ti,ab. |
| 20 | (stability or interrater or inter-rater or intrarater or intra-rater or intertester or inter-tester or intratester or intra-tester or interobserver or inter-observer or intraobserver or intra-observer or intertechnician or inter-technician or intratechnician or intra-technician or interexaminer or inter-examiner or intraexaminer or intra-examiner or interassay or inter-assay or intraassay or intra-assay or interindividual or inter-individual or intraindividual or intra-individual or interparticipant or inter-participant or intraparticipant or intra-participant or kappa or kappa's or kappas or repeatab*).ti,ab. |
| 21 | ((replicab* or repeated) and (measure or measures or findings or result or results or test or tests)).ti,ab. |
| 22 | (generaliza* or generalisa* or concordance).ti,ab. |
| 23 | (intraclass and correlation*).ti,ab. |
| 24 | (multitrait and scaling and (analysis or analyses)).ti,ab. |
| 25 | (item discriminant or interscale correlation* or error or errors or "individual variability").ti,ab. |
| 26 | (variability and (analysis or values)).ti,ab. |
| 27 | (uncertainty and (measurement or measuring)).ti,ab. |
| 28 | ("standard error of measurement" or sensitiv* or responsive*).ti,ab. |
| 29 | ((minimal or minimally or clinical or clinically) and (important or significant or detectable) and (change or difference)).ti,ab. |
| 30 | (discriminative or "known group" or factor analysis or factor analyses or dimension* or subscale*).ti,ab. |
| 31 | (small* and (real or detectable) and (change or difference)).ti,ab. |
| 32 | (meaningful change or "ceiling effect" or "floor effect" or "Item response model" or IRT or Rasch or "Differential item functioning" or DIF or "computer adaptive testing" or "item bank" or "cross-cultural equivalence").ti,ab. |
| 33 | 1 or 2 or 3 or 4 or 5 or 6 or 7 or 8 or 9 or 10 or 11 or 12 or 13 or 14 or 15 or 16 or 17 or 18 or 19 or 20 or 21 or 22 or 23 or 24 or 25 or 26 or 27 or 28 or 29 or 30 or 31 or 32 |
| 34 | Primary Sclerosing Cholangitis.mp. or exp Cholangitis, Sclerosing/ |
| 35 | sclerosing cholangitis.tw. |
| 36 | (Primary sclerosing Cholangitis or PSC).tw. |
| 37 | cholangiocarcinoma.mp. or exp cholangiocarcinoma/ |
| 38 | cholangiocarcinoma.tw. |
| 39 | cholestasis.mp. or cholestasis.tw. |
| 40 | cholestasis/ |
| 41 | 34 or 35 or 36 or 37 or 38 or 39 or 40 |
| 42 | "outcome assessment (Health Care)"/ |
| 43 | tool*.ti,ab. |
| 44 | instrument*.ti,ab. |
| 45 | questionnaire*.ti,ab. |
| 46 | index.ti,ab. |
| 47 | indices.ti,ab. |
| 48 | scale*.ti,ab. |
| 49 | survey*.ti,ab. |
| 50 | feedback.ti,ab. |
| 51 | interview*.ti,ab. |
| 52 | (outcome* adj2 measure*).ti,ab. |
| 53 | (outcome* adj2 assessment*).ti,ab. |
| 54 | PROMS.ti,ab. |
| 55 | (measur* adj2 (quality or health or outcomes)).ti,ab. |
| 56 | (assess* adj2 (quality or health or outcomes)).ti,ab. |
| 57 | (patient report* adj2 outcome* adj2 (measure* or assessment*)).ti,ab. |
| 58 | (self report* adj2 outcome* adj2 (measure* or assessment*)).ti,ab. |
| 59 | (self assess* adj2 outcome* adj2 (measure* or assessment*)).ti,ab. |
| 60 | "quality of life"/ |
| 61 | HRQOL.ti,ab. |
| 62 | QOL.ti,ab. |
| 63 | QL.ti,ab. |
| 64 | HRQL.ti,ab. |
| 65 | health utilit*.ti,ab. |
| 66 | health outcomes.ti,ab. |
| 67 | patient outcome*.ti,ab. |
| 68 | (patient reported adj2 outcome*).ti,ab. |
| 69 | (self reported adj2 outcome*).ti,ab. |
| 70 | (patient assessed adj2 outcome*).ti,ab. |
| 71 | (self assessed adj2 outcome*).ti,ab. |
| 72 | ((health or functional) adj status).ti,ab. |
| 73 | (well being or wellbeing).ti,ab. |
| 74 | functioning.ti,ab. |
| 75 | activit*.ti,ab. |
| 76 | participation.ti,ab. |
| 77 | Patient reported outcome*.tw. |
| 78 | Self reported outcome*.tw. |
| 79 | exp "Quality of Life"/ |
| 80 | Patient Satisfaction/ |
| 81 | adherence.mp. |
| 82 | Fatigue/ |
| 83 | exp *Health Status/ |
| 84 | "Activities of Daily Living"/ |
| 85 | life qualit$.tw. |
| 86 | exp self concept/ |
| 87 | health level.tw. |
| 88 | level of health.tw. |
| 89 | wellness.tw. |
| 90 | well being.tw. |
| 91 | (activities of daily life or daily living activities).tw. |
| 92 | functional ability.tw. |
| 93 | good health.tw. |
| 94 | healthiness.tw. |
| 95 | social adjustment/ |
| 96 | physical limitations.tw. |
| 97 | psychiatric status.tw. |
| 98 | pain measurement/ |
| 99 | functional assessment.tw. |
| 100 | exp *"Outcome Assessment (Health Care)"/ |
| 101 | health status.tw. |
| 102 | lifestyle.tw. |
| 103 | questionnaire*.tw. |
| 104 | symptom assessment.tw. |
| 105 | needs assessment.tw. |
| 106 | quality of life.tw. |
| 107 | exp *Questionnaires/ |
| 108 | self report$.tw. |
| 109 | patient$ experience$.tw. |
| 110 | PROM$1.tw. |
| 111 | *Pain/ |
| 112 | Pain, Postoperative/ |
| 113 | *"Severity of Illness Index"/ |
| 114 | Health Utility.tw. |
| 115 | psychosocial.tw. |
| 116 | Patient Satisfaction.mp. or exp Patient Satisfaction/ |
| 117 | (outcome$ adj5 expectation$).tw. |
| 118 | exp Interviews as Topic/ |
| 119 | (symptom$ and (improv$ or change$ or deteriorat$)).ti,ab. |
| 120 | (patient$ and priorit$).ti,ab. |
| 121 | (scale or scales).ti,ab. |
| 122 | expectations.tw. |
| 123 | satisfaction.tw. |
| 124 | treatment satisfaction.tw. |
| 125 | Recovery of Function.tw. |
| 126 | capabilit$.tw. |
| 127 | Quality of life.ti,ab. |
| 128 | psychological adapt$.tw. |
| 129 | 42 or 43 or 44 or 45 or 46 or 47 or 48 or 49 or 50 or 51 or 52 or 53 or 54 or 55 or 56 or 57 or 58 or 59 or 60 or 61 or 62 or 63 or 64 or 65 or 66 or 67 or 68 or 69 or 70 or 71 or 72 or 73 or 74 or 75 or 76 or 77 or 78 or 79 or 80 or 81 or 82 or 83 or 84 or 85 or 86 or 87 or 88 or 89 or 90 or 91 or 92 or 93 or 94 or 95 or 96 or 97 or 98 or 99 or 100 or 101 or 102 or 103 or 104 or 105 or 106 or 107 or 108 or 109 or 110 or 111 or 112 or 113 or 114 or 115 or 116 or 117 or 118 or 119 or 120 or 121 or 122 or 123 or 124 or 125 or 126 or 127 or 128 |
| 130 | 41 and 129 |
| 131 | 33 and 130 |
