# Supplementary material for: Patient-reported outcome measures used in patients with primary sclerosing cholangitis: a systematic review
Source: Health Qual Life Outcomes. 2018 Jul 5;16:133. doi: 10.1186/s12955-018-0951-6 (PMC6034220; doi:10.1186/s12955-018-0951-6)
Supplement: Supplementary file 3 — Content comparison. (DOCX 52 kb) [file 12955_2018_951_MOESM3_ESM.docx]

**Additional file 3 Content Comparison**

| **Table 3: Content comparison of included PROMs** | | | | | | | | | | | | | | | | | |
| --- | --- | --- | --- | --- | --- | --- | --- | --- | --- | --- | --- | --- | --- | --- | --- | --- | --- |
| **Domains** | **15 D** | **SF-36** | **SF-6D** | **LDQOL** | **CLDQ** | **SIBDQ** | **FIS** | **NIDDK-QA** | **PGWB** | **BDI** | **GSRS** | **PedsQL** | **EQ-5D** | **FFSS** | **MFI** | **PBC-27** | **PBC-40** |
| Symptoms | ✓ |  |  |  |  |  |  |  |  |  |  |  |  |  |  | ✓ | ✓ |
| Liver disease related symptoms |  |  |  | ✓ |  |  |  | ✓ |  |  |  |  |  |  |  |  |  |
| Effect of liver disease |  |  |  | ✓ |  |  |  |  |  |  |  |  |  |  |  |  |  |
| Abdominal symptoms |  |  |  |  | ✓ |  |  |  |  |  |  |  |  |  |  |  |  |
| Abdominal pain symptoms |  |  |  |  |  |  |  |  |  |  |  |  |  |  |  |  |  |
| Abdominal pain syndrome |  |  |  |  |  |  |  |  |  |  | ✓ |  |  |  |  |  |  |
| Systemic symptoms |  |  |  |  | ✓ | ✓ |  |  |  |  |  |  |  |  |  |  |  |
| Bowel symptoms |  |  |  |  |  | ✓ |  |  |  |  |  |  |  |  |  |  |  |
| Indigestion syndrome |  |  |  |  |  |  |  |  |  |  | ✓ |  |  |  |  |  |  |
| Reflux syndrome |  |  |  |  |  |  |  |  |  |  | ✓ |  |  |  |  |  |  |
| Diarrhoea syndrome |  |  |  |  |  |  |  |  |  |  | ✓ |  |  |  |  |  |  |
| Constipation syndrome |  |  |  |  |  |  |  |  |  |  | ✓ |  |  |  |  |  |  |
| Endogenous symptoms |  |  |  |  |  |  |  |  |  |  |  |  |  |  |  |  |  |
| Urgency |  |  |  |  |  |  |  |  |  |  |  |  |  |  |  |  |  |
| Oesophageal symptoms |  |  |  |  |  |  |  |  |  |  |  |  |  |  |  |  |  |
| Gastroduodenal symptoms |  |  |  |  |  |  |  |  |  |  |  |  |  |  |  |  |  |
| Biliary symptoms |  |  |  |  |  |  |  |  |  |  |  |  |  |  |  |  |  |
| Bowel movements |  |  |  |  |  |  |  |  |  |  |  |  |  |  |  |  |  |
| Anorectal symptoms |  |  |  |  |  |  |  |  |  |  |  |  |  |  |  |  |  |
| Soiling / seepage |  |  |  |  |  |  |  |  |  |  |  |  |  |  |  |  |  |
| Perinatal / stomal soreness |  |  |  |  |  |  |  |  |  |  |  |  |  |  |  |  |  |
| Orthostatic Intolerance |  |  |  |  |  |  |  |  |  |  |  |  |  |  |  |  |  |
| Evaluation difficulties |  |  |  |  |  |  |  |  |  |  |  |  |  |  |  |  |  |
| Vasomotor |  |  |  |  |  |  |  |  |  |  |  |  |  |  |  |  |  |
| Secretomotor |  |  |  |  |  |  |  |  |  |  |  |  |  |  |  |  |  |
| Gastrointestinal |  |  |  |  |  |  |  |  |  |  |  |  |  |  |  |  |  |
| Dryness |  |  |  |  |  |  |  |  |  |  |  |  |  |  |  | ✓ |  |
| Itch/ Pruritus |  |  |  |  |  |  |  |  |  |  |  |  |  |  |  | ✓ | ✓ |
| Fatigue |  |  |  |  | ✓ |  |  |  |  |  |  |  |  | ✓ |  | ✓ | ✓ |
| General fatigue |  |  |  |  |  |  |  |  |  |  |  |  |  |  | ✓ |  |  |
| Physical fatigue |  |  |  |  |  |  |  |  |  |  |  |  |  |  | ✓ |  |  |
| Mental fatigue |  |  |  |  |  |  |  |  |  |  |  |  |  |  | ✓ |  |  |
| Reduced motivation |  |  |  |  |  |  |  |  |  |  |  |  |  |  | ✓ |  |  |
| Reduced activity |  |  |  |  |  |  |  |  |  |  |  |  |  |  | ✓ |  |  |
|  |  |  |  |  |  |  |  |  |  |  |  |  |  |  |  |  |  |
| Pain |  |  | ✓ |  |  |  |  |  |  |  |  |  | ✓ |  |  |  |  |
| Bodily pain |  | ✓ |  | ✓ |  |  |  |  |  |  |  |  |  |  |  |  |  |
| Discomfort | ✓ |  |  |  |  |  |  |  |  |  |  |  | ✓ |  |  |  |  |
| Abdominal pain syndrome |  |  |  |  |  |  |  |  |  |  |  |  |  |  |  |  |  |
|  |  |  |  |  |  |  |  |  |  |  |  |  |  |  |  |  |  |
| Physical |  |  |  |  |  |  |  |  |  |  |  |  |  |  |  |  |  |
| Physical role |  | ✓ |  | ✓ |  |  |  |  |  |  |  |  |  |  |  |  |  |
| Physical functioning |  | ✓ | ✓ | ✓ |  |  | ✓ |  |  |  |  | ✓ |  |  |  |  |  |
| Role limitation |  |  | ✓ |  |  |  |  |  |  |  |  |  |  |  |  |  |  |
| Mobility | ✓ |  |  |  |  |  |  |  |  |  |  |  | ✓ |  |  |  |  |
| Vision | ✓ |  |  |  |  |  |  |  |  |  |  |  |  |  |  |  |  |
| Hearing | ✓ |  |  |  |  |  |  |  |  |  |  |  |  |  |  |  |  |
| Breathing | ✓ |  |  |  |  |  |  |  |  |  |  |  |  |  |  |  |  |
| Sleeping | ✓ |  |  | ✓ |  |  |  |  |  |  |  |  |  |  |  |  |  |
| Speech | ✓ |  |  |  |  |  |  |  |  |  |  |  |  |  |  |  |  |
| Eating | ✓ |  |  |  |  |  |  |  |  |  |  |  |  |  |  |  |  |
| Elimination | ✓ |  |  |  |  |  |  |  |  |  |  |  |  |  |  |  |  |
| Dietary restriction |  |  |  |  |  |  |  |  |  |  |  |  |  |  |  |  |  |
| Activities | ✓ |  |  |  | ✓ |  |  |  |  |  |  |  |  |  |  |  |  |
| Self-care |  |  |  |  |  |  |  |  |  |  |  |  | ✓ |  |  |  |  |
| School functioning |  |  |  |  |  |  |  |  |  |  |  | ✓ | ✓ |  |  |  |  |
|  |  |  |  |  |  |  |  |  |  |  |  |  |  |  |  |  |  |
| Social |  |  |  |  |  |  |  |  |  |  |  |  |  |  |  | ✓ | ✓ |
| Social functioning |  | ✓ | ✓ | ✓ |  | ✓ |  |  |  |  |  | ✓ |  |  |  |  |  |
| Social handicap |  |  |  |  |  |  |  |  |  |  |  |  |  |  |  |  |  |
|  |  |  |  |  |  |  |  |  |  |  |  |  |  |  |  |  |  |
| Emotion |  |  |  |  |  |  |  |  |  |  |  |  |  |  |  | ✓ | ✓ |
| Emotional role |  | ✓ |  | ✓ |  |  |  |  |  |  |  |  |  |  |  |  |  |
| Emotional function |  |  |  |  | ✓ | ✓ |  |  |  |  |  |  |  |  |  |  |  |
| Loneliness |  |  |  | ✓ |  |  |  |  |  |  |  | ✓ |  |  |  |  |  |
| Stigma of liver disease |  |  |  | ✓ |  |  |  |  |  |  |  |  |  |  |  |  |  |
|  |  |  |  |  |  |  |  |  |  |  |  |  |  |  |  |  |  |
| Psychological |  |  |  |  |  |  |  |  |  |  |  |  |  |  |  |  |  |
| Psychosocial functioning |  |  |  |  |  |  | ✓ | ✓ |  |  |  |  |  |  |  |  |  |
| Mental health |  | ✓ | ✓ | ✓ |  |  |  |  |  |  |  |  |  |  |  |  |  |
| Mental function | ✓ |  |  |  |  |  |  |  |  |  |  |  |  |  |  |  |  |
| Distress | ✓ |  |  |  |  |  |  |  |  |  |  |  |  |  |  |  |  |
| Health distress |  |  |  | ✓ |  |  |  |  |  |  |  |  |  |  |  |  |  |
| Concentration/ memory |  |  |  | ✓ |  |  |  |  |  |  |  |  |  |  |  |  |  |
| Worry |  |  |  |  | ✓ |  |  |  |  |  |  |  |  |  |  |  |  |
| Anxiety |  |  |  |  |  |  |  |  | ✓ |  |  |  | ✓ |  |  |  |  |
| Cognitive |  |  |  |  |  |  |  |  |  |  |  |  |  |  |  | ✓ | ✓ |
| Cognitive functioning |  |  |  |  |  |  | ✓ |  |  |  |  |  |  |  |  |  |  |
| Cognitive changes |  |  |  |  |  |  |  |  |  |  |  |  |  |  |  |  |  |
| Cognitive-affective |  |  |  |  |  |  |  |  |  | ✓ |  |  |  |  |  |  |  |
| Depression | ✓ |  |  |  |  |  |  |  |  |  |  |  | ✓ |  |  |  |  |
| Depression mood |  |  |  |  |  |  |  |  | ✓ |  |  |  |  |  |  |  |  |
| Depressive syndrome |  |  |  |  |  |  |  |  |  |  |  |  |  |  |  |  |  |
| Somatic symptoms |  |  |  |  |  |  |  |  |  | ✓ |  |  |  |  |  |  |  |
| Self-control |  |  |  |  |  |  |  |  | ✓ |  |  |  |  |  |  |  |  |
| Mood disturbance |  |  |  |  |  |  |  |  |  |  |  |  |  |  |  |  |  |
| Suicidal ideation & behaviour |  |  |  |  |  |  |  |  |  |  |  |  |  |  |  |  |  |
| Endogenous symptoms |  |  |  |  |  |  |  |  |  |  |  |  |  |  |  |  |  |
| Hopelessness |  |  |  | ✓ |  |  |  |  |  |  |  |  |  |  |  |  |  |
| Vegetative features |  |  |  |  |  |  |  |  |  |  |  |  |  |  |  |  |  |
| Mood Disturbance |  |  |  |  |  |  |  |  |  |  |  |  |  |  |  |  |  |
|  |  |  |  |  |  |  |  |  |  |  |  |  |  |  |  |  |  |
| Sexual activity | ✓ |  |  |  |  |  |  |  |  |  |  |  |  |  |  |  |  |
| Sexual functioning / problems |  |  |  | ✓ |  |  |  |  |  |  |  |  |  |  |  |  |  |
| Sexual desire |  |  |  |  |  |  |  |  |  |  |  |  |  |  |  |  |  |
| Arousal |  |  |  |  |  |  |  |  |  |  |  |  |  |  |  |  |  |
| Lubrication |  |  |  |  |  |  |  |  |  |  |  |  |  |  |  |  |  |
| Orgasm |  |  |  |  |  |  |  |  |  |  |  |  |  |  |  |  |  |
| Orgasmic function |  |  |  |  |  |  |  |  |  |  |  |  |  |  |  |  |  |
| Erectile function |  |  |  |  |  |  |  |  |  |  |  |  |  |  |  |  |  |
| Intercourse satisfaction |  |  |  |  |  |  |  |  |  |  |  |  |  |  |  |  |  |
|  |  |  |  |  |  |  |  |  |  |  |  |  |  |  |  |  |  |
| General health |  | ✓ |  | ✓ |  |  |  |  | ✓ |  |  |  |  |  |  |  |  |
| Vitality | ✓ | ✓ | ✓ | ✓ |  |  |  |  | ✓ |  |  |  |  |  |  |  |  |
| Overall well-being |  |  |  |  |  |  |  |  | ✓ |  |  |  |  |  |  |  |  |
| Positive well-being |  |  |  |  |  |  |  | ✓ |  |  |  |  |  |  |  |  |  |
| Overall QoL & general health status |  |  |  |  |  |  |  |  |  |  |  |  |  |  |  |  |  |
|  |  |  |  |  |  |  |  |  |  |  |  |  |  |  |  |  |  |
| Global/ Overall satisfaction |  |  |  |  |  |  |  |  |  |  |  |  |  |  |  |  |  |
| Health Satisfaction |  |  |  |  |  |  |  | ✓ |  |  |  |  |  |  |  |  |  |
| Environment |  |  |  |  |  |  |  |  |  |  |  |  |  |  |  |  |  |
|  | | | | | | | | | | | | | | |  |  |  |
| **Domains** | **WHOQOL-BREF** | **IDS** | **FSFI** | **IIEFI** | **Roman II Modular** | **OS** | **HADS** | **SADS** | **PSC PRO** | **5-D Itch** |  |  |  |  |  |  |  |
| Symptoms |  |  |  |  |  |  |  |  | ✓ | ✓ |  |  |  |  |  |  |  |
| Liver related symptoms |  |  |  |  |  |  |  |  |  |  |  |  |  |  |  |  |  |
| Effect of liver disease |  |  |  |  |  |  |  |  |  |  |  |  |  |  |  |  |  |
| Abdominal symptoms |  |  |  |  |  |  |  |  |  |  |  |  |  |  |  |  |  |
| Abdominal pain symptoms |  |  |  |  | ✓ |  |  |  | ✓ |  |  |  |  |  |  |  |  |
| Abdominal pain syndrome |  |  |  |  |  |  |  |  |  |  |  |  |  |  |  |  |  |
| Systemic symptoms |  |  |  |  |  |  |  |  |  |  |  |  |  |  |  |  |  |
| Bowel symptoms |  |  |  |  | ✓ |  |  |  |  |  |  |  |  |  |  |  |  |
| Indigestion syndrome |  |  |  |  |  |  |  |  |  |  |  |  |  |  |  |  |  |
| Reflux syndrome |  |  |  |  |  |  |  |  |  |  |  |  |  |  |  |  |  |
| Diarrhoea syndrome |  |  |  |  |  |  |  |  |  |  |  |  |  |  |  |  |  |
| Constipation syndrome |  |  |  |  |  |  |  |  |  |  |  |  |  |  |  |  |  |
| Endogenous symptoms |  |  |  |  |  |  |  |  |  |  |  |  |  |  |  |  |  |
| Urgency |  |  |  |  |  | ✓ |  |  |  |  |  |  |  |  |  |  |  |
| Esophageal symptoms |  |  |  |  | ✓ |  |  |  |  |  |  |  |  |  |  |  |  |
| Gastroduodenal symptoms |  |  |  |  | ✓ |  |  |  |  |  |  |  |  |  |  |  |  |
| Biliary symptoms |  |  |  |  | ✓ |  |  |  |  |  |  |  |  |  |  |  |  |
| Bowel movements |  |  |  |  |  | ✓ |  |  |  |  |  |  |  |  |  |  |  |
| Anorectal symptoms |  |  |  |  | ✓ |  |  |  |  |  |  |  |  |  |  |  |  |
| Soiling / seepage |  |  |  |  |  | ✓ |  |  |  |  |  |  |  |  |  |  |  |
| Perinatal / stomal soreness |  |  |  |  |  | ✓ |  |  |  |  |  |  |  |  |  |  |  |
| Orthostatic Intolerance |  |  |  |  |  |  |  |  |  |  |  |  |  |  |  |  |  |
| Evaluation difficulties |  |  |  |  |  | ✓ |  |  |  |  |  |  |  |  |  |  |  |
| Vasomotor |  |  |  |  |  |  |  |  |  |  |  |  |  |  |  |  |  |
| Secretomotor |  |  |  |  |  |  |  |  |  |  |  |  |  |  |  |  |  |
| Gastrointestinal |  |  |  |  |  |  |  |  |  |  |  |  |  |  |  |  |  |
| Dryness |  |  |  |  |  |  |  |  |  |  |  |  |  |  |  |  |  |
| Itch/ Pruritus |  |  |  |  |  |  |  |  | ✓ | ✓ |  |  |  |  |  |  |  |
| Fatigue |  |  |  |  |  |  |  |  |  |  |  |  |  |  |  |  |  |
| General fatigue |  |  |  |  |  |  |  |  |  |  |  |  |  |  |  |  |  |
| Physical fatigue |  |  |  |  |  |  |  |  | ✓ |  |  |  |  |  |  |  |  |
| Mental fatigue |  |  |  |  |  |  |  |  | ✓ |  |  |  |  |  |  |  |  |
| Reduced motivation |  |  |  |  |  |  |  |  |  |  |  |  |  |  |  |  |  |
| Reduced activity |  |  |  |  |  |  |  |  |  |  |  |  |  |  |  |  |  |
|  |  |  |  |  |  |  |  |  | ✓ |  |  |  |  |  |  |  |  |
| Pain |  |  | ✓ |  |  |  |  |  |  |  |  |  |  |  |  |  |  |
| Bodily pain |  |  |  |  |  |  |  |  |  |  |  |  |  |  |  |  |  |
| Discomfort |  |  |  |  |  |  |  |  |  |  |  |  |  |  |  |  |  |
| Abdominal pain syndrome |  |  |  |  |  |  |  |  |  |  |  |  |  |  |  |  |  |
|  |  |  |  |  |  |  |  |  |  |  |  |  |  |  |  |  |  |
| Physical | ✓ |  |  |  |  |  |  |  | ✓ |  |  |  |  |  |  |  |  |
| Physical role |  |  |  |  |  |  |  |  |  |  |  |  |  |  |  |  |  |
| Physical functioning |  |  |  |  |  |  |  |  | ✓ |  |  |  |  |  |  |  |  |
| Role limitation |  |  |  |  |  |  |  |  |  |  |  |  |  |  |  |  |  |
| Mobility |  |  |  |  |  |  |  |  |  |  |  |  |  |  |  |  |  |
| Vision |  |  |  |  |  |  |  |  |  |  |  |  |  |  |  |  |  |
| Hearing |  |  |  |  |  |  |  |  |  |  |  |  |  |  |  |  |  |
| Breathing |  |  |  |  |  |  |  |  |  |  |  |  |  |  |  |  |  |
| Sleeping |  |  |  |  |  |  |  |  |  |  |  |  |  |  |  |  |  |
| Speech |  |  |  |  |  |  |  |  |  |  |  |  |  |  |  |  |  |
| Eating |  |  |  |  |  |  |  |  |  |  |  |  |  |  |  |  |  |
| Elimination |  |  |  |  |  |  |  |  |  |  |  |  |  |  |  |  |  |
| Dietary restriction |  |  |  |  |  | ✓ |  |  |  |  |  |  |  |  |  |  |  |
| Activities |  |  |  |  |  |  |  |  | ✓ |  |  |  |  |  |  |  |  |
| Self-care |  |  |  |  |  |  |  |  | ✓ |  |  |  |  |  |  |  |  |
| School functioning |  |  |  |  |  |  |  |  |  |  |  |  |  |  |  |  |  |
|  |  |  |  |  |  |  |  |  |  |  |  |  |  |  |  |  |  |
| Social |  |  |  |  |  |  |  |  |  |  |  |  |  |  |  |  |  |
| Social relationship | ✓ |  |  |  |  |  |  |  |  |  |  |  |  |  |  |  |  |
| Social functioning |  |  |  |  |  |  |  |  |  |  |  |  |  |  |  |  |  |
| Social handicap |  |  |  |  |  | ✓ |  |  |  |  |  |  |  |  |  |  |  |
|  |  |  |  |  |  |  |  |  |  |  |  |  |  |  |  |  |  |
| Emotion |  |  |  |  |  |  |  |  |  |  |  |  |  |  |  |  |  |
| Emotional role |  |  |  |  |  |  |  |  |  |  |  |  |  |  |  |  |  |
| Emotional function |  |  |  |  |  |  |  |  |  |  |  |  |  |  |  |  |  |
| Loneliness |  |  |  |  |  |  |  |  |  |  |  |  |  |  |  |  |  |
| Stigma of liver disease |  |  |  |  |  |  |  |  |  |  |  |  |  |  |  |  |  |
|  |  |  |  |  |  |  |  |  |  |  |  |  |  |  |  |  |  |
| Psychological | ✓ |  |  |  |  |  |  |  |  |  |  |  |  |  |  |  |  |
| Psychosocial functioning |  |  |  |  |  |  |  |  |  |  |  |  |  |  |  |  |  |
| Mental health |  |  |  |  |  |  |  |  |  |  |  |  |  |  |  |  |  |
| Mental function |  |  |  |  |  |  |  |  |  |  |  |  |  |  |  |  |  |
| Distress |  |  |  |  |  |  |  |  |  |  |  |  |  |  |  |  |  |
| Health distress |  |  |  |  |  |  |  |  |  |  |  |  |  |  |  |  |  |
| Concentration/ memory |  |  |  |  |  |  |  |  | ✓ |  |  |  |  |  |  |  |  |
| Worry |  |  |  |  |  |  |  |  | ✓ |  |  |  |  |  |  |  |  |
| Anxiety |  | ✓ |  |  |  |  | ✓ |  |  |  |  |  |  |  |  |  |  |
| Cognitive |  |  |  |  |  |  |  |  |  |  |  |  |  |  |  |  |  |
| Cognitive functioning |  |  |  |  |  |  |  |  |  |  |  |  |  |  |  |  |  |
| Cognitive changes |  | ✓ |  |  |  |  |  |  |  |  |  |  |  |  |  |  |  |
| Cognitive-affective |  |  |  |  |  |  |  |  | ✓ |  |  |  |  |  |  |  |  |
| Depression |  |  |  |  |  |  | ✓ |  |  |  |  |  |  |  |  |  |  |
| Depressive mood and ideation |  |  |  |  |  |  |  | ✓ |  |  |  |  |  |  |  |  |  |
| Depressive syndrome |  |  |  |  |  |  |  | ✓ |  |  |  |  |  |  |  |  |  |
| Somatic symptoms |  |  |  |  |  |  |  |  |  |  |  |  |  |  |  |  |  |
| Self-control |  |  |  |  |  |  |  |  |  |  |  |  |  |  |  |  |  |
| Mood disturbance |  |  |  |  |  |  |  |  |  |  |  |  |  |  |  |  |  |
| Suicidal ideation & behaviour |  |  |  |  |  |  |  | ✓ |  |  |  |  |  |  |  |  |  |
| Endogenous symptoms |  | ✓ |  |  |  |  |  | ✓ |  |  |  |  |  |  |  |  |  |
| Hopelessness |  |  |  |  |  |  |  |  |  |  |  |  |  |  |  |  |  |
| Vegetative features |  | ✓ |  |  |  |  |  |  |  |  |  |  |  |  |  |  |  |
| Mood Disturbance |  | ✓ |  |  |  |  |  |  |  |  |  |  |  |  |  |  |  |
|  |  |  |  |  |  |  |  |  |  |  |  |  |  |  |  |  |  |
| Sexual activity |  |  |  |  |  |  |  |  | ✓ |  |  |  |  |  |  |  |  |
| Sexual functioning / problems |  |  |  |  |  |  |  |  |  |  |  |  |  |  |  |  |  |
| Sexual desire |  |  | ✓ | ✓ |  |  |  |  |  |  |  |  |  |  |  |  |  |
| Arousal |  |  | ✓ |  |  |  |  |  |  |  |  |  |  |  |  |  |  |
| Lubrication |  |  | ✓ |  |  |  |  |  |  |  |  |  |  |  |  |  |  |
| Orgasm |  |  | ✓ |  |  |  |  |  |  |  |  |  |  |  |  |  |  |
| Orgasmic function |  |  |  | ✓ |  |  |  |  |  |  |  |  |  |  |  |  |  |
| Erectile function |  |  |  | ✓ |  |  |  |  |  |  |  |  |  |  |  |  |  |
| Intercourse satisfaction |  |  |  | ✓ |  |  |  |  |  |  |  |  |  |  |  |  |  |
| General health |  |  |  |  |  |  |  |  |  |  |  |  |  |  |  |  |  |
| Vitality |  |  |  |  |  |  |  |  |  |  |  |  |  |  |  |  |  |
| Positive well-being |  |  |  |  |  |  |  |  |  |  |  |  |  |  |  |  |  |
| Overall QoL & general health status | ✓ |  |  |  |  |  |  |  |  |  |  |  |  |  |  |  |  |
|  |  |  |  |  |  |  |  |  |  |  |  |  |  |  |  |  |  |
| Global/ Overall satisfaction |  |  | ✓ | ✓ |  |  |  |  |  |  |  |  |  |  |  |  |  |
| Health Satisfaction |  |  |  |  |  |  |  |  | ✓ |  |  |  |  |  |  |  |  |
| Environment | ✓ |  |  |  |  |  |  |  |  |  |  |  |  |  |  |  |  |
|  | | | | | | | | | | | | | | | | | |
