# Supplementary material for: Patient-reported outcome measures used in patients with primary sclerosing cholangitis: a systematic review
Source: Health Qual Life Outcomes. 2018 Jul 5;16:133. doi: 10.1186/s12955-018-0951-6 (PMC6034220; doi:10.1186/s12955-018-0951-6)
Supplement: Supplementary file 4 — Cosmin checklist. (DOCX 22 kb) [file 12955_2018_951_MOESM4_ESM.docx]

**Additional file 4 COSMIN CHECKLIST**

**STUDY NAME:** Kim et al. 2000

Step 1: Evaluated measurement properties in the study by Kim et al. 2000

- Internal consistency
- Reliability
- Hypothesis testing
- Criterion Validity

Step 2: Skipped IRT model not used

**Measurement properties**

**Internal consistency**

1. Does the scale consist of effect indicators, i.e. is it based on a reflective model?

*Design requirements*

1. Was the percentage of missing items given?

Answer: Good- Percentage of missing items NOT described

1. Was there a description of how missing items were handled?

Answer: Fair- Not clear how missing items were handled

1. Was the sample size included in the internal consistency analysis adequate?

Answer: Poor - Small sample size (<30)

1. Was unidimensionality of the scale checked? i.e. was factor analysis or IRT model applied?

Answer: Poor – factor analysis NOT performed and no reference to another study

1. Was the sample size included in the unidimensionality analysis adequate?

Answer: Unidimensionality analysis was not conducted by the authors

1. Was an internal consistency statistic calculated for each (unidimensional) (sub) scale separately?

Answer: Excellent - Internal consistency statistic calculated

1. Were there any important flaws in the design or methods of the study?

Answer: Fair- Other minor methodological flaws in the design or execution of the study

*Statistical methods*

1. For Classical Test Theory (CTT), continuous scores: was Cronbach’s alpha calculated?

Answer: Excellent- Cronbach’s alpha or KR-20 calculated

1. For CTT, dichotomous scores: Was Cronbach’s alpha or KR-20 calculated?

Answer: Not applicable

1. For IRT: was a goodness of fit statistic at a global level calculated? E.g. X^2^, reliability coefficient of estimated latent trait values (index of ( subject of item) separation)

Answer: Not applicable

**Reliability: relative measures (including test-retest reliability, inter-rater reliability, inter- rater reliability and intra-rater reliability)**

*Design requirements*

1. Was the percentage of missing items given?

Answer: Good - Percentage of missing items NOT described

1. Was there a description of how missing items were handled?

Answer: Fair - Not clear how missing items were handled

1. Was the sample size included in the analysis adequate?

Answer: Poor - Small sample size (<30)

1. Were at least two measurements available?

Answer: Excellent - At least two measurements

1. Were the administrations independent?

Answer: Excellent - Independent measurements

1. Was the time interval stated?

Answer: Excellent - Time interval

1. Were patients stable in the interim period on the construct to be measured?

Answer: Good - Assumable that patients were stable

1. Was the time interval appropriate?

Answer: Excellent - Time interval appropriate

1. Were the test conditions similar for both measurements? e.g. type of administration, environment, instructions

Answer: Good - assumable that test conditions were similar

1. Were there any important flaws in design or methods of the study?

Answer: Fair - Other minor methodological flaws in the design or execution of the study

*Statistical methods*

1. For continuous scores: Was an intraclass correlation coefficient (ICC) calculated?

Answer: Fair - Pearson or spearman correlation coefficient calculated WITHOUT evidence provided that systematic change has occurred or WITH evidence that systematic change has occurred

1. For dichotomous/nominal/ordinal scores: was kappa calculated?

Answer: Not applicable

1. For ordinal scores: Was a weighted kappa calculated?

Answer: Not applicable

1. For ordinal scores: Was the weighting scheme described? e.g. linear, quadratic

Answer: Not applicable

**Hypotheses testing**

*Design requirements*

1. Was the percentage of missing items given?

Answer: Good - Percentage of missing items NOT described

1. Was there a description of how missing items were handled?

Answer: Fair - Not clear how missing items were handled

1. Was the sample size included in the analysis adequate?

Answer: Poor - Small sample size (<30)

1. Were hypotheses regarding correlations or mean differences formulated a priori (i.e. before data collection?

Answer: Poor - Unclear what was expected

1. Was the expected direction of correlations or mean differences included in the hypotheses?

Answer: Good - Expected direction of the correlations or differences NOT stated

1. Was the expected absolute or relative magnitude of correlations or mean differences included in the hypotheses?

Answer: Good - Expected magnitude of the correlations or differences NOT stated

1. For convergent validity: Was an adequate description provided of the comparator instrument(s)?

Answer: Excellent - Adequate description of the constructs measured by the comparator instruments (s).

1. For convergent validity: Were the measurement properties of the comparator instrument(s) adequately described?

Answer: Poor - No information on the measurement properties of the comparator instrument.

1. Were any important flaws in design or methods of the study?

Answer: Fair - Other minor methodological flaws the design or execution of the study (e.g. only data presented on a comparison with an instrument that measure another construct).

*Statistical methods*

1. Were design and statistical methods adequate for the hypotheses to be tested?

Answer: Assumable that statistical methods were appropriate e.g. Pearson correlations applied, but distribution of scores or mean (SD) not presented.

**Criterion validity**

*Design requirements*

1. Was the percentage of missing items given?

Answer: Good - Percentage of missing items NOT described

2. Was there a description of how missing items were handled?

Answer: Fair - Not clear how missing items were handled

3. Was the sample size included in the analysis adequate?

Answer: Poor - Small sample size (<30)

4. Can the criterion used or employed be considered as a reasonable ‘gold standard’?

Answer: Poor - Criterion used can NOT be considered an adequate ‘gold standard’.

5. Were there any important flaws in the design or methods of the study?

Answer: Fair - Other minor methodological flaws in the design or execution of the study

*Statistical methods*

6. For continuous scores: Were correlations or the area under the receiver operating curve calculated?

Answer: Excellent – Correlations or AUC calculated

7. For dichotomous scores: Were sensitivity and specificity determined?

Answer: Not applicable

**STUDY NAME:** Gross et al. 1999

Step 1: Evaluated measurement properties in the study by Gross et al. 1999

- Internal consistency

Step 2: Skipped IRT model not used

**Measurement properties**

**Internal consistency**

1. Does the scale consist of effect indicators, i.e. is it based on a reflective model?

*Design requirements*

1. Was the percentage of missing items given?

Answer: Good- Percentage of missing items NOT described

1. Was there a description of how missing items were handled?

Answer: Fair- Not clear how missing items were handled

1. Was the sample size included in the internal consistency analysis adequate?

Answer: Good - Good sample size (50-99)

1. Was unidimensionality of the scale checked? i.e. was factor analysis or IRT model applied?

Answer: Poor - factor analysis NOT performed and no reference to another study

1. Was the sample size included in the unidimensionality analysis adequate?

Answer: Unidimensionality analysis was not conducted

1. Was an internal consistency statistic calculated for each (unidimensional) (sub) scale separately?

Answer: Poor - Internal consistency statistic NOT calculated for each subscale seperately

1. Were there any important flaws in the design or methods of the study?

Answer: Poor - Other important methodological flaws in the design or execution of the study

*Statistical methods*

1. For Classical Test Theory (CTT), continuous scores: was Cronbach’s alpha calculated?

Answer: Excellent- Cronbach’s alpha or KR-20 calculated

1. For CTT, dichotomous scores: Was Cronbach’s alpha or KR-20 calculated?

Answer: Not applicable

1. For IRT: was a goodness of fit statistic at a global level calculated? E.g. X^2^, reliability coefficient of estimated latent trait values (index of ( subject of item) separation)

Answer: Not applicable
